# Unraveling the Immune Web: Advances in SMI Capsular Fibrosis from Molecular Insights to Preclinical Breakthroughs

**DOI:** 10.3390/biom14111433

**Published:** 2024-11-11

**Authors:** Ines Schoberleitner, Klaus Faserl, Michaela Lackner, Débora C. Coraça-Huber, Angela Augustin, Anja Imsirovic, Stephan Sigl, Dolores Wolfram

**Affiliations:** 1 Institute of Pathology, Neuropathology and Molecular Pathology, Medical University of Innsbruck, Müllerstraße 44, 6020 Innsbruck, Austria; 2 Department of Plastic, Reconstructive and Aesthetic Surgery, Medical University of Innsbruck, Anichstraße 35, 6020 Innsbruck, Austria; 3 Protein Core Facility, Institute of Medical Chemistry, Biocenter, Medical University of Innsbruck, Innrain 80-82, 6020 Innsbruck, Austria; 4 Institute of Hygiene and Medical Microbiology, Medical University of Innsbruck, Schöpfstraße 41, 6020 Innsbruck, Austria; 5 BIOFILM Lab, Department of Orthopedics and Traumatology, Medical University of Innsbruck, Müllerstraße 44, 6020 Innsbruck, Austria; 6 Department of Obstetrics and Gynecology, Medical University of Innsbruck, Anichstraße 35, 6020 Innsbruck, Austria

**Keywords:** silicone mammary implants (SMIs), immune mechanisms, inflammatory response, capsular fibrosis, immunomics, biofilm formation, biomaterial innovations, immunomodulatory surfaces, personalized medicine, therapeutic targets

## Abstract

Breast implant surgery has evolved significantly, yet challenges such as capsular contracture remain a persistent concern. This review presents an in-depth analysis of recent advancements in understanding the immune mechanisms and clinical implications associated with silicone mammary implants (SMIs). The article systematically examines the complex interplay between immune responses and capsular fibrosis, emphasizing the pathophysiological mechanisms of inflammation in the etiology of this fibrotic response. It discusses innovations in biomaterial science, including the development of novel anti-biofilm coatings and immunomodulatory surfaces designed to enhance implant integration and minimize complications. Emphasis is placed on personalized risk assessment strategies, leveraging molecular insights to tailor interventions and improve patient outcomes. Emerging therapeutic targets, advancements in surgical techniques, and the refinement of post-operative care are also explored. Despite notable progress, challenges such as the variability in immune responses, the long-term efficacy of new interventions, and ethical considerations remain. Future research directions are identified, focusing on personalized medicine, advanced biomaterials, and bridging preclinical findings with clinical applications. As we advance from bench to bedside, this review illuminates the path forward, where interdisciplinary collaboration and continued inquiry weave together to enhance the art and science of breast implant surgery, transforming patient care into a realm of precision and excellence.

## 1. Introduction

Inspired by “genomics”, various “-omics” fields such as transcriptomics, proteomics, metabolomics, glycomics, and lipidomics have emerged over the past two decades [1,2]. Traditional biochemical methods are inefficient, while omics technologies use high-throughput methods such as microarrays and mass spectrometry to generate extensive datasets [1,3,4]. Supported by bioinformatics, these advancements offer significant insights into biological mechanisms, forming a comprehensive framework for modern life science [5,6,7,8].

The postgenomic era has driven omics technologies in biomedical [2,6,9,10,11] and pharmaceutical research [8,12], enabling efficient exploration of genomes, transcriptomes, and proteomes with high sensitivity and resolution [13]. These advancements facilitate the identification of therapeutic targets, drug safety assessments, and molecular-based diagnostics, paving the way for personalized healthcare [7,9]. Omics technologies revolutionize biological research by examining collective interactions within cellular systems or biochemical processes rather than isolated components. Omics represents the evolution of collective thought and data, forming a crucial part of systems biology [2,7,9,12].

Systems immunology offers a comprehensive understanding of the immune system by examining single immunological components and pathways as interconnected networks [14]. This holistic approach contrasts with methods that focus on individual parts, aiming instead to elucidate how these parts interact and function together, a complex task requiring specialized methodologies [15]. Over the past century, experimental strategies have defined cell types and states within the immune system, revealing key molecular and functional components and establishing causal relationships in the transcriptional and functional cascades driving immune activation [16].

High-throughput, high-resolution technologies from the omics field have revolutionized immunology in the last two decades, enabling the simultaneous assessment of numerous cellular, functional, and molecular parameters [15,17]. Immunomics, intersecting immunology and genomics, is crucial in studying implant-based capsular fibrosis, particularly with SMIs [18,19,20]. The complex interplay between the immune system and foreign materials leads to adverse reactions and fibrotic responses [21,22,23,24,25,26,27,28,29,30].

Silicone, widely used in medical implants, triggers foreign body responses (FBRs), leading to the formation of a fibrous capsule and impaired tissue function [31,32,33,34,35]. While silicone mammary implants (SMIs) have transformed breast augmentation and reconstruction [36,37,38], their clinical utility is often hampered by capsular contracture, characterized by excessive extracellular matrix (ECM) accumulation around the implant [39]. This fibrotic reaction, driven by the host immune response, presents a significant clinical challenge [40].

The initiation of fibrosis involves an inflammatory phase mediated by innate and adaptive immune cells [28,29,30,41]. Macrophages, neutrophils, and mast cells activate fibroblasts to produce ECM proteins [18,20,28,29,30,41,42,43,44,45]. Silicone binds non-specifically to blood proteins, leading to inflammation and protein adsorption on implants’ surfaces [30,41,46,47,48,49,50]. Macrophages and other immune cells uptake silicone debris, contributing to fibrotic responses [48,51,52,53,54,55]. SMIs exemplify foreign body-induced fibrotic diseases that are relevant to other silicone-based medical devices [30,31,32,41,55,56,57].

In recent years, there has been a growing interest in elucidating the molecular mechanisms underlying SMI-associated capsular fibrosis [28,29,45,58], with a particular focus on the intricate interplay between the immune system and implanted materials [34,41,54,55,56,57,59,60,61,62,63]. Understanding these immune-mediated processes is crucial for developing targeted therapeutic strategies to mitigate or prevent capsular fibrosis and enhance patient outcomes following breast implant surgery.

This review provides a comprehensive overview of the immune-mediated mechanisms involved in SMI-associated capsular fibrosis, focusing on molecular insights and potential therapeutic interventions. We explore the molecular pathways and cellular responses that drive fibrosis in response to SMI implantation. Additionally, we discuss the impact of capsular fibrosis on patient outcomes, diagnostic challenges, and current treatment modalities.

Preclinical breakthroughs and innovative research strategies aimed at understanding SMI-induced fibrosis highlight their translational potential for developing targeted therapies. Our goal is to provide a thorough understanding of SMI-associated capsular fibrosis and its immune-mediated pathogenesis, identifying novel therapeutic avenues to improve outcomes in breast implant surgery.

Immunomics offers significant promise for understanding the immunological basis of implant-based capsular fibrosis (IBCF) and devising innovative diagnostic and treatment strategies. By modulating immune responses and inflammation pathways identified through immunomics, tailored interventions can prevent or alleviate capsular contracture. Through interdisciplinary collaboration and cutting-edge technologies, immunomics has the potential to revolutionize the management of capsular fibrosis in implant-based surgeries.

## 2. Molecular Mechanisms of SMI-Associated Capsular Fibrosis

Breast augmentation with SMIs is a widely practiced procedure, with approximately 2.2 million surgeries performed in 2022 alone [64]. Despite its popularity, peri-SMI capsular contracture remains a significant complication, with reported incidence rates ranging from 0.5% to 50% [65,66,67,68]. This condition involves the formation of a fibrous capsule around the implant, leading to pain, aesthetic issues, and potential functional impairment, necessitating revision surgery or even implant removal.

In contrast, breast reconstruction is often performed after a mastectomy and typically employs an immediate two-stage expander-based approach. This method involves the use of an inflatable implant known as a tissue expander, which allows for the gradual expansion of the mastectomy skin flap before the placement of a permanent implant [69,70,71]. The choice of tissue expander, influenced by surface roughness, plays a critical role in determining the final aesthetic outcomes and the likelihood of fibrotic capsule formation [39,55,63,72,73,74,75]. Histopathological studies underscore that the type of tissue expander used can imprint characteristics onto the capsule, influencing the long-term results [39,41,57,62].

Capsular fibrosis results from a complex interplay of factors, primarily driven by the FBR initiated upon implantation [28]. This cascade involves excessive deposition of collagenous and non-collagenous ECM components by activated fibroblasts and myofibroblasts. Chronic inflammatory reactions triggered by stimuli such as infections, autoimmune reactions, and tissue injury further exacerbate fibrosis through pathways including TGF-β, Smad, NF-κB, and MAPK signaling [19,21,22,23,24,25,26,27,34].

Research on silicone-induced fibrosis, particularly with SMIs, serves as a model for understanding foreign body-induced fibrotic diseases.

SMIs trigger a complex immune response that leads to fibrosis [30,41]. Understanding this immune-mediated process is crucial, as it significantly influences the success and durability of the implant [39]. The body’s reaction to SMIs involves several intertwined cellular and molecular events, including inflammation, immune cell activation, and the formation of fibrotic tissue.

**Figure 1:**
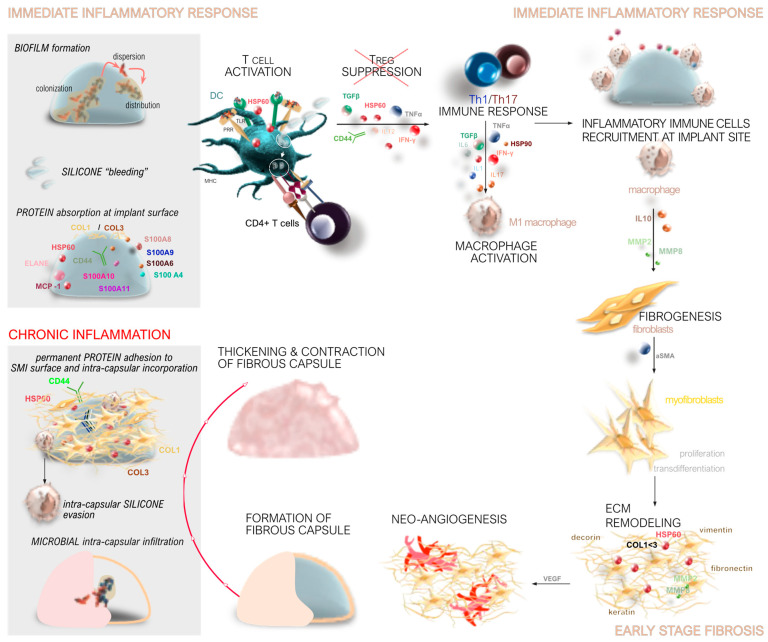
Stages of capsular fibrosis around SMIs. *Immediate wound response*. During the acute wound healing phase, immediately after SMI implantation, the implant is exposed to wound bed fluid. T cells are activated primarily due to microbial contamination, implant shedding or silicone bleeding, and protein adsorption onto the implant’s surface and differentiate into Th1 and Th17 responses, while the T regulatory (Treg) response is suppressed. *Foreign body response*. Innate immune cells, such as neutrophils, macrophages, and dendritic cells, are recruited to the implant site. Macrophages are activated and play a key role in fibrogenesis, contributing to the early stages of fibrosis. *Early-stage fibrosis* The extracellular matrix undergoes remodeling with significant collagen deposition. The fibrotic tissue encapsulating the implant undergoes neo-angiogenesis, leading to the formation of a fibrous capsule. *Chronic inflammation and capsular contracture*. Chronic inflammation is perpetuated by the permanent adhesion of proteins to the SMI’s surface. Intracapsular silicone evasion and microbial infiltration further exacerbate the inflammatory response. This leads to excessive ECM remodeling and collagen deposition. The resulting thickened and contracted capsule causes discomfort and pain to the patient, potentially leading to implant displacement and necessitating revision surgery.

The development of capsular fibrosis around SMIs follows a well-defined sequence of biological events. This process begins with immediate immune activation, followed by early inflammatory and fibrotic changes. Over time, chronic inflammation can develop, ultimately leading to the formation of a fibrous capsule and, in some cases, capsular contracture (Figure 1).

When an SMI is implanted, the body recognizes it as a foreign object, triggering an immediate immune response (Figure 1). This response begins with the activation of the innate immune system, which includes the recruitment of immune cells such as neutrophils, macrophages, and dendritic cells to the site of SMI implantation. These cells release cytokines and chemokines, signaling molecules that mediate inflammation and recruit additional immune cells to the area [28,29,30,41,76,77].

During the acute phase of the immune response, which occurs within the first few days post-implantation, there is a surge in systemic inflammatory mediators. This includes a rapid increase in cytokines such as IFN-γ, IL-1β, and TNF-α, which are associated with a Th1-type immune response [28,29,41]. These cytokines promote inflammation and prepare the site for the subsequent wound healing and fibrosis formation phases [30].

Macrophages play a crucial role in the immune response to SMIs (Figure 1). They can adopt different functional phenotypes, including the pro-inflammatory M1 phenotype and the anti-inflammatory, tissue-remodeling M2 phenotype. The balance between these phenotypes influences the extent of inflammation and fibrosis around the implant [30,41,63].

M1 macrophages, which are predominant during the early inflammatory response, produce pro-inflammatory cytokines that exacerbate inflammation. In contrast, M2 macrophages, which appear later, secrete anti-inflammatory cytokines and growth factors that promote tissue repair and fibrosis [41,78]. The persistence of M1 macrophages and a delayed transition to the M2 phenotype can lead to chronic inflammation and excessive fibrosis [41,63,78].

Fibrosis around SMIs is a result of the body’s attempt to isolate and protect itself from the foreign material. This process involves the deposition of ECM components (Figure 1), such as collagen, which form a fibrous capsule around the implant [30,77,79,80]. The degree of fibrosis is influenced by various factors, including the extent of the initial inflammatory response and the duration of chronic inflammation [30,41,77,79,80].

Key mediators of fibrosis include transforming growth factor-beta (TGF-β), which stimulates the production of ECM components and inhibits their degradation [28,29,30,41,77]. The balance between matrix metalloproteinases (MMPs), which degrade ECM, and their tissue inhibitors (TIMPs) is also crucial in regulating fibrosis [28,29,30,41,77]. An imbalance favoring TIMPs over MMPs can lead to excessive ECM deposition and fibrosis [41,79,80].

Chronic inflammation is a significant factor in the long-term response to SMIs (Figure 1). Persistent inflammation can result in ongoing tissue remodeling and fibrosis, leading to the formation of a thick, fibrous capsule around the implant. This capsule can contract over time, causing capsular contracture, which is a common complication of breast implants [32,39,76,81,82].

Markers of chronic inflammation, such as S100 proteins, are often up-regulated in response to SMIs. These proteins play a role in sustaining the inflammatory response and promoting fibrosis [30,41,83,84,85,86]. The accumulation of pro-inflammatory and pro-fibrotic cytokines, such as IL-17 and TGF-β, further contributes to chronic inflammation and fibrosis [30,41,63,78].

The extent of capsular contracture varies based on factors such as implant type [87,88,89,90]—specifically, whether they are saline-filled or silicone gel-filled [87,88], as well as the surface characteristics (smooth or textured) [89,90,91]—and implant position, which can be categorized as subglandular or subpectoral [87,92]. Each of these variables plays a unique role in modulating the body’s immune response, tissue integration, and, consequently, the likelihood of capsular contracture.

Silicone gel-filled implants, for instance, tend to have a slightly different immune response profile compared with saline-filled implants. The viscosity and cohesive nature of the silicone gel can lead to different mechanical interactions with the surrounding tissues, which may impact capsular contracture rates by influencing how tissue fibers organize around the implant [87,88].

Clinical studies have provided compelling evidence that implant surfaces with smoother textures, specifically those with reduced roughness profiles such as Ra 4 µm, elicit reduced levels of inflammation and fibrosis compared with their rougher counterparts [30,39,41,55,59,63,93,94]. These findings underscore the pivotal role of surface microtopography in modulating tissue responses post-implantation. Smoother surfaces are associated with diminished immune activation, characterized by lower levels of pro-inflammatory cytokines and a decelerated pro-fibrotic response [41,55,63]. This effect is attributed to altered protein adsorption patterns and reduced cell adhesion on smoother surfaces, which collectively contribute to a more favorable tissue integration environment [30,41,56,59,61]. By minimizing the inflammatory cascade and promoting a balanced cytokine profile, smoother surfaces have the potential to enhance implant biocompatibility and mitigate complications such as capsular contracture [39,41]. These insights highlight the significance of surface engineering strategies aimed at optimizing implant design to improve patient outcomes across diverse clinical settings.

In recent years, breast implant-associated anaplastic large cell lymphoma (BIA-ALCL) has emerged as a critical concern in breast implant surgery, prompting heightened awareness and regulatory scrutiny [76]. This rare type of lymphoma is associated primarily with textured breast implants and may arise from chronic inflammatory responses and immunological reactions to the implants’ surface [76]. Although BIA-ALCL is not classified as breast cancer, it poses significant health risks and necessitates careful monitoring and patient education. Understanding the immunological dynamics surrounding BIA-ALCL is vital, as it influences the choice of implant materials and design, aiming to minimize long-term complications and optimize patient outcomes.

In addition to implant type, the expander used in the initial reconstruction stage significantly impacts long-term outcomes [39,93]. Studies have demonstrated that variations in silicone implants’ surface topographies significantly affect the final reconstructive outcomes by influencing tissue imprinting. One investigation assessed 12 different SMIs to identify their impact on capsular contracture. The findings indicated that different surface textures resulted in notable differences in capsular thickness, inflammatory cell counts, and collagen expression. Specifically, smooth-textured implants such as SEBBIN and Mentor exhibited higher collagen formation and a reduction in inflammatory cytokines (IL-8, CD68, MCP-1, and F4/80) compared with textured implants, suggesting that smoother surfaces may mitigate inflammatory responses and subsequent fibrotic encapsulation [95]. Further supporting this, another study conducted an intraindividual analysis comparing micro-textured expanders (Ra 4 µm) with rougher alternatives (Ra 60 µm) in patients undergoing breast reconstruction. This research found that smoother implant surfaces, characterized by their reduced roughness, resulted in significantly lower capsular thickness and improved biocompatibility [39].

Implant position also plays a significant role in capsular contracture outcomes. When implants are positioned subglandularly (above the pectoral muscle), there is often greater exposure to breast tissue, which can increase the inflammatory response and the likelihood of contracture. Conversely, subpectoral placement (beneath the pectoral muscle) may provide a degree of protection, potentially reducing the mechanical strain on the implant and dampening inflammatory signaling [87,92].

These findings highlight the crucial role of surface roughness in modulating the host response and achieving better cosmetic outcomes.

## 3. Genomics and Proteomics in SMI-Associated Capsular Fibrosis

### 3.1. Genomic Insights into SMI-Associated Capsular Fibrosis

Genomic profiling techniques have been instrumental in uncovering the molecular basis of capsular fibrosis, a major complication following breast implantation surgeries. A recent genome-wide study has provided significant insights into the genetic landscape underlying SMI-associated capsular fibrosis.

#### 3.1.1. Genomic Profiling of Capsular Fibrosis

In their seminal study, Kyle et al. employed whole-genome transcriptome analysis of capsular tissue to explore the dysregulated genetic landscape associated with breast fibrotic capsule formation, shedding light on potential biomarkers that govern this pathological process [96]. By leveraging microarray technology, the researchers identified 257 significantly dysregulated genes in contracted breast capsules compared with the controls. From these, six genes were scrutinized further on the basis of their biological relevance and degree of dysregulation: aggrecan (ACAN), interleukin-8 (IL-8), matrix metallopeptidase 12 (MMP12), serum amyloid A 1 (SAA1), tissue inhibitor of metalloproteinase 4 (TIMP4), and tumor necrosis factor superfamily member 11 (TNFSF11). The findings underscored distinct patterns of gene expression in contracted capsules, revealing up-regulation of IL-8, MMP12, and SAA1, and down-regulation of ACAN, TIMP4, and TNFSF11. Validation through quantitative reverse transcriptase polymerase chain reaction (QRT-PCR) corroborated these findings, confirming significant up-regulation of IL-8 and down-regulation of ACAN, TIMP4, and TNFSF11 in contracted capsules. Immunohistochemistry (IHC) further supported these results, demonstrating increased protein expression of IL-8 and MMP12 alongside decreased expression of TIMP4 in contracted tissues [96].

**Figure 2:**
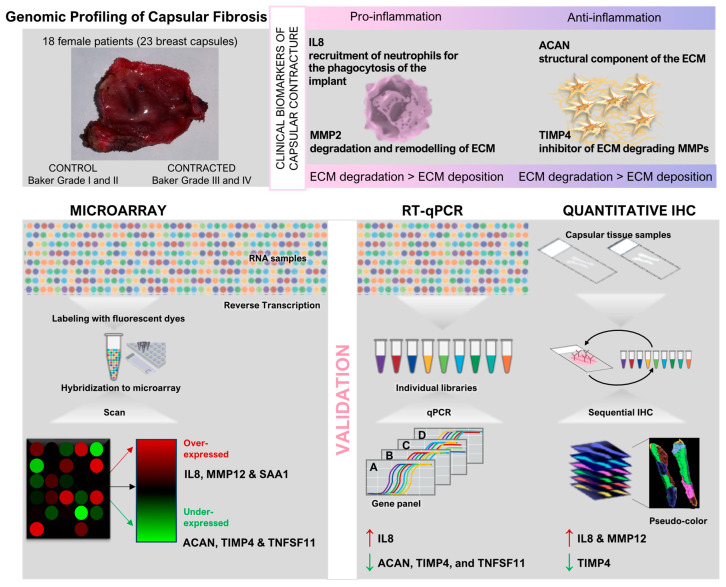
Genomic profiling of capsular fibrosis around SMIs. Comprehensive profiling of capsular fibrosis around SMIs through genomic techniques has elucidated the underlying molecular mechanisms and potential clinical applications. *Clinical context:* The study involved 18 female patients with 23 breast capsules, comparing control (Baker Grade I and II) and contracted (Baker Grade III and IV) capsules. *Genomic techniques:* RNA samples underwent labeling with fluorescent dyes, reverse transcription, hybridization to microarray, and scanning to identify over-expressed (IL-8, MMP12, SAA1) and under-expressed (ACAN, TIMP4, TNFSF11) genes. *Validation:* qPCR and sequential immunohistochemistry (IHC) validated the expression of key genes, with IL-8 and MMP12 showing over-expression and ACAN, TIMP4, and TNFSF11 showing under-expression. *Pro- and anti-inflammatory genes:* The highlighted genes include pro-inflammatory markers (IL-8, MMP2) associated with ECM degradation and anti-inflammatory markers (ACAN, TIMP4) associated with ECM deposition.

As detailed in Figure 2, genome-wide studies represent a pivotal advancement in the exploration of SMI capsular fibrosis, providing insights into the intricate molecular mechanisms underlying this complex condition.

The study’s [96] approach not only highlighted the pivotal role of inflammatory responses (IL-8) and extracellular matrix remodeling (MMP12) in capsular fibrosis but also emphasized the potential diagnostic and therapeutic implications of these findings. IL-8, known for its role in acute inflammation and fibrotic conditions, emerged as a key mediator in perpetuating the FBR around breast implants. Conversely, TIMP4’s diminished expression suggested a dysregulated matrix remodeling environment conducive to fibrotic encapsulation.

However, while RT-qPCR offers high sensitivity and specificity in quantifying gene expression, its scope is often limited to known targets and predefined pathways, potentially overlooking novel genes that are crucial to the disease process [96,97]. In contrast, RNA Seq presents a promising alternative by enabling unbiased, genome-wide profiling of gene expression, thus facilitating the discovery of novel biomarkers and pathways implicated in SMI-associated capsular fibrosis, as already demonstrated for other types of inflammatory, fibrotic diseases affecting soft tissues, including the lung, liver, kidney, and skin [98].

This methodological shift from targeted approaches such as RT-qPCR to RNA Seq not only enhances the breadth of data captured but also supports the identification of previously unrecognized molecular signatures driving disease progression. Despite these advantages, RNA Seq is not without challenges, including the need for substantial bioinformatics expertise and computational resources for data analysis, which may pose barriers to its widespread adoption in clinical settings.

#### 3.1.2. Challenges and Future Directions

Applying genomic profiling techniques provides a comprehensive understanding of the molecular mechanisms underpinning breast capsular contracture formation. By identifying novel biomarkers and unraveling intricate gene expression patterns, this research establishes a foundational basis for developing future diagnostic tools and targeted therapeutic strategies to reduce the occurrence and severity of this prevalent post-surgical complication. It is important to note that a significant challenge in advancing genomic research on SMI-associated capsular fibrosis is the acquisition of comprehensive patient material. Variability in disease presentation, patient demographics, and disease severity necessitate large-scale, well-characterized cohorts for robust statistical analyses and generalizability of the findings [99,100,101,102].

### 3.2. Proteomic Insights into SMI-Associated Capsular Fibrosis

Proteomics differs from genomic studies by directly investigating the protein composition, modifications, and interactions that define the pathophysiology of capsular fibrosis. This capability is essential for identifying specific biomarkers reflecting disease progression and treatment responses, enhancing diagnostic accuracy and prognostic assessment in clinical settings. By uncovering novel protein biomarkers and pathways associated with SMI-associated capsular fibrosis, proteomics not only deepens our understanding of disease mechanisms but also establishes the groundwork for personalized therapeutic strategies aimed at improving patient care and outcomes. Analyzing SMI-associated capsular fibrosis through proteomics rather than genomics provides distinct advantages due to its ability to capture dynamic protein interactions at the implant interface and the surrounding tissues, which play crucial roles in inflammation, immune responses, and fibrotic changes.

#### 3.2.1. Proteomic Studies on Protein Adsorption and Immune Responses

Protein adsorption to the surface of SMIs has been extensively studied, both by incubation with serum proteins [56,61,62,103] and post-operatively by stripping off the proteome in animal models [63]. The use of silicone-linked immunosorbent assay (SILISA) facilitated an understanding of how proteins adhere to silicone implants, inducing adverse immunological reactions and fibrotic responses [56,61]. Statistical analyses of proteins such as fibronectin, C-reactive protein (CRP), immunoglobulin G (IgG), and heat shock protein 60 (HSP 60) revealed their significant correlation with fibrotic reactions.

In another study, researchers investigated serological parameters in 143 individuals, including 93 with SMIs and 50 controls, to evaluate the systemic effects associated with these implants [62]. Patients with SMIs exhibited elevated levels of circulating immune complexes (CIC), anti-polymer antibodies (APA), procollagen III, and soluble intercellular adhesion molecule-1 (sICAM-1) compared with the controls. These differences correlated with the severity of capsular fibrosis and the duration of implantation, indicating a connection between serological abnormalities and fibrotic complications surrounding SMIs. The study underscored the potential clinical utility of these serological markers in predicting and monitoring adverse outcomes in SMI patients, advocating for thorough clinical and serologic monitoring in this population.

Of note, the serum is not the only origin of FBR toward implants. Tissue injury, after surgical insertion of SMIs, immediately activates the innate immune system, setting in motion a local inflammatory response and pro-inflammatory mediation that includes the recruitment of inflammatory cells from the circulation [28,29]. Nowadays, wound fluid is commonly used for protein profiling and analysis. However, the correct method of sample collection is crucial in highly sensitive proteomic analyses [104,105].

#### 3.2.2. Proteomic Profiling of SMI Surfaces

Protein adsorption to silicone surfaces was further investigated using an untargeted proteomics approach focused on identifying proteins critical for local immune reactions to silicone implants [103]. The study utilized both in vivo analyses of explanted silicone implants and in vitro models incubated with wound bed fluid. Differential analysis, mass spectrometry, database matching, and Western blotting were employed to identify the 30 most abundant proteins adhering to silicone (e.g., actin, fibronectin, vitronectin, fibrinogen, collagen I, laminin, and MMP2). Structural proteins, host defense mediators, and transport-related proteins emerged as the predominant components. Additionally, biochemical modifications of fibronectin, vitronectin, and HSP 60 were observed post-adhesion. These findings underscored the role of the silicone surface´s properties in protein degradation and unfolding, potentially leading to immune responses and fibrotic processes. New therapeutic targets identified include fibronectin and vitronectin, which play critical roles in mediating inflammatory responses and fibrotic processes, as well as MMP2, an enzyme involved in extracellular matrix remodeling that could be targeted to prevent or reduce capsular contracture. For untargeted proteomics and biomarker discovery studies, identification and measurement of large numbers of proteins simultaneously by mass spectrometry is favored, yet rare in implant immunoreactivity research [106].

#### 3.2.3. Intraindividual Comparative Proteomic Profiling

**Figure 3:**
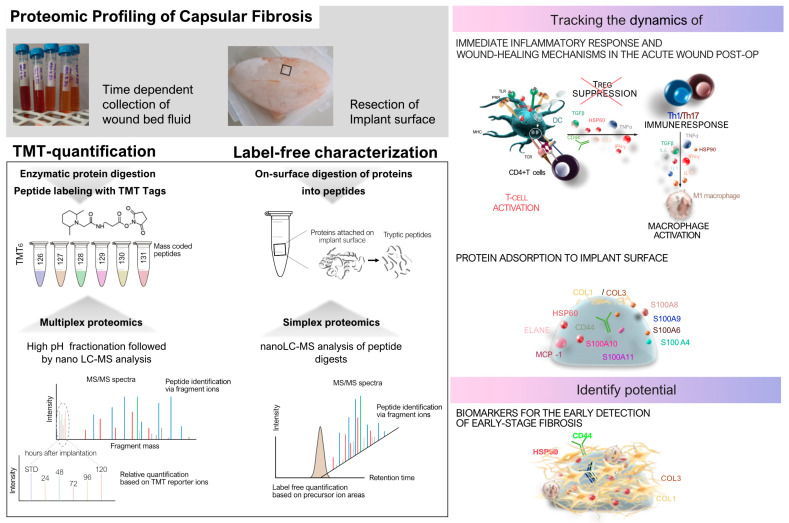
Proteomic profiling of capsular fibrosis around SMIs. Comprehensive profiling of capsular fibrosis around SMIs through proteomic techniques, elucidating the underlying molecular mechanisms and potential clinical applications. *Clinical context:* this study comprised 10 female patients who underwent simultaneous prophylactic bilateral nipple-sparing mastectomy (NSME) and tissue expander-based breast reconstruction. Biological samples of wound bed fluid (referred to as WBF) were collected daily from Day 1 to 5 following the expander’s implantation. Wound drains, integral to the surgical procedure for patients undergoing expander-based reconstruction, were retained post-operatively. WBF was collected under sterile conditions. During reoperation, capsular tissue was harvested from two different implants. *Proteomic techniques:* Time-dependent collection of wound bed fluid and resection of the implant surface followed by enzymatic protein digestion and peptide labeling with TMT tags. *Multiplexed proteomics:* High pH fractionation, nano-LC-MS analysis of peptide digests, and on-surface digestion of proteins into peptides revealed time-dependent protein expression and quantification post-surgery. Integration and clinical implications. *Immediate inflammatory response:* This depicts the dynamics of the inflammatory response and wound healing post-implantation, including T cell activation, Th1/Th17 immune response, T regulatory (Treg) cell suppression, and macrophage activation. *Protein adsorption and progression of fibrosis:* Continuous protein adsorption to the implants’ surface and ECM remodeling contributed to capsular contracture and fibrosis. *Biomarkers for early detection:* Identified potential biomarkers such as HSPs and S100 proteins for early-stage fibrosis, offering diagnostic and therapeutic insights.

Protein adsorption and its implications were further investigated in real time in patients through intraindividual comparative proteomic profiling of plasma, wound fluid, and adhesive peptidomes associated with SMIs over an extended period post-SMI implantation (Figure 3) [30]. Pre-operative plasma samples were taken before surgery to understand the baseline systemic protein profiles. In addition, wound fluid was collected daily from Day 1 to Day 5 post-implantation from surgical drains to capture the acute phase of wound healing and inflammation. Approximately eight months after implantation, proteins adhered to the SMIs’ surface were analyzed to identify chronic inflammatory responses and potential biomarkers for capsular fibrosis.

By utilizing samples from the same individuals, the study minimized interindividual variability and provided a comprehensive characterization of protein profile changes during different phases of wound healing and inflammatory reactions at the implant site. Proteins from both wound fluid and plasma samples were quantified using tandem mass tags TMT-based mass spectrometry. Furthermore, proteins that adhered to the silicone surface of the tissue expanders (inflatable SMIs), which were removed about six to eight months post-implantation, were analyzed using nano-LC-MS/MS.

The intraindividual comparative proteomic profiling of plasma, wound fluid, and adhesive proteomes associated with SMIs provided a detailed insight into the dynamic changes in protein levels during the post-operative healing process and the subsequent development of fibrosis. The pre-operative plasma samples established a systemic protein baseline, while the daily post-operative wound fluid samples captured the acute immune response to tissue injury and implant placement. The proteomic landscape during the early post-operative period showed elevated expression of proteins involved in oxidative stress, coagulation, and immediate wound healing, such as superoxide dismutase, catalase, and plasminogen activator inhibitor-1 (PAL-1), which are critical for controlling bleeding and clot formation. The wound fluid also revealed neutrophil-derived enzymes such as matrix metalloproteinases (MMP8, MMP9), underscoring the onset of ECM remodeling—a process crucial for both normal wound healing and the progression of fibrosis.

At eight months post-implantation, the adhesive proteome on the SMIs’ surface reflected a shift towards chronic inflammation, with the persistence of proteins linked to ECM turnover, such as various collagen types (COL1, COL3, COL6) and fibrosis drivers such as S100A8 and S100A9, indicating an ongoing fibrotic response. Antimicrobial proteins such as PGLYRP1 and CAMP were also present, suggesting a sustained immune response contributing to chronic inflammation. Heat shock proteins (HSP60, HSP90) adhered to the implants’ surface, playing roles in both inflammatory regulation and fibrosis. These findings point to a prolonged immune reaction and ECM dysregulation, further driving fibrotic encapsulation around the implant.

In comparison with genomic profiling [96], this method provided a more direct view of the actual protein activities and changes at the implant site over time. Genomic analyses tend to highlight potential signaling pathways and gene expression, such as those mediated by TGF-β and interferon-γ, which modulate fibroblast activation and ECM deposition. However, proteomic profiling revealed the functional protein products and their interactions within the wound environment, offering a clearer picture of the real-time biological processes. While genomic data emphasize regulatory cytokines such as IL-6 and TNF-α in the transition from inflammation to fibrosis, proteomic profiling directly captures the proteins driving ECM remodeling and chronic immune responses, offering potential biomarkers for diagnosing and managing capsular fibrosis in breast implant patients.

This method enabled a detailed investigation of the wound proteome’s response to different surface textures of SMIs [30,41,59].

### 3.3. Integration of Genomics and Proteomics for Comprehensive Understanding

#### 3.3.1. Complementary Roles of Genomics and Proteomics

Genomic profiling techniques, such as RT-qPCR and RNA Seq, have shed light on the molecular mechanisms underpinning conditions such as capsular fibrosis, emphasizing the critical pathways related to inflammation and ECM remodeling. By identifying key differentially expressed genes, these analyses elucidate how various cellular signals contribute to the fibrotic process [96,97,98].

In contrast, proteomic approaches provide a real-time perspective by examining protein interactions directly at the implant interface [30,41,61]. Techniques such as SILAC [61] and mass spectrometry [30,41] allow for the detection of specific proteins involved in chronic inflammation and the development of fibrosis, offering a dynamic view of the biological landscape. These proteomic studies have identified novel biomarkers that could enhance diagnostic accuracy and inform therapeutic strategies for managing capsular fibrosis.

While genomic analyses focus on gene expression patterns, proteomic studies capture the temporal changes in protein expression and their contributions to inflammatory responses and the progression of fibrosis. Together, these findings illuminate the complex interplay between molecular and protein-level changes, highlighting potential biomarkers for early detection and identifying new therapeutic targets. This integrative approach promises to improve clinical outcomes for patients with SMI by providing a comprehensive understanding of the mechanisms driving fibrotic encapsulation.

#### 3.3.2. Future Directions and Clinical Implications

The integration of genomic and proteomic approaches is essential for advancing our understanding of the complex molecular mechanisms underlying SMI-associated capsular fibrosis. While genomic profiling provides critical insights into the gene expression patterns and underlying genetic factors, proteomics offers a dynamic perspective by directly analyzing protein interactions and their roles in disease pathogenesis.

Combining these methodologies not only enriches understanding of the fibrotic process but also enhances the potential for identifying novel biomarkers for early detection and targeted therapies. As research progresses, leveraging both genomic and proteomic data will enable the development of personalized medicine strategies tailored to individual patient profiles.

Future studies should focus on integrating multi-omics data to elucidate the interplay between genetic variations and protein expression in the context of capsular fibrosis. This holistic approach can reveal critical pathways involved in inflammation and fibrosis development, potentially leading to the discovery of new therapeutic targets.

The integration of interdisciplinary research and cutting-edge technologies is expected to enhance clinical outcomes for individuals undergoing SMI-based breast reconstruction and augmentation, including those seeking aesthetic enhancements, by facilitating the development of personalized treatment strategies informed by comprehensive biological insights.

## 4. Fibroblast Dynamics and Immune Interactions: Navigating Capsular Fibrosis in Silicone Implant Biocompatibility

### 4.1. Fibroblast Activation and Differentiation

Capsular fibrosis, a common complication following silicone implantation, involves complex interactions between the biomaterial’s surfaces and host tissue responses, prominently featuring fibroblast activation and differentiation processes [28]. Fibroblasts are crucial in wound healing and tissue repair, undergoing distinct activation states in response to signals from the implant microenvironment [107].

#### 4.1.1. Role of Fibroblasts in Wound Healing

Fibroblasts play a vital role in all three phases of wound healing (WH), orchestrating the repair process by producing regulatory molecules and interacting with other cell populations involved in healing mechanisms [108,109]. Injury triggers an inflammatory reaction via cytokines from platelet degranulation [110]. Immune cells increase pro-inflammatory mediators, such as interleukin-1 (IL-1), interleukin-6 (IL-6), interleukin-12 (IL-12), tumor necrosis factor-α (TNF-α), and inducible nitric oxide synthase (iNOS), fueling inflammation and stimulating fibroblast recruitment and activation [110].

#### 4.1.2. Fibroblasts’ Response to Silicone Implants

Silicone surfaces initiate a cascade of events triggering acute and chronic inflammatory responses [28,29,30]. Initial acute responses involve immune cell recruitment and cytokine release, influencing fibroblasts’ behavior [28,29,30]. Immunohistochemical analysis of fibrous capsules from patients with SMIs showed the presence of fibroblasts along with macrophages, dendritic cells, and activated CD4+ T cells at the capsule–silicone implant interface [57]. Activated fibroblasts transition into myofibroblasts, characterized by α-smooth muscle actin (α-SMA) expression and enhanced contractility [111]. Fibroblasts accumulate around implants, correlating with the severity of contracture as per the Baker classification system [112,113]. This indicates fibroblasts play a crucial role in the formation and maintenance of the fibrotic capsule.

#### 4.1.3. Crosstalk and Inflammatory Phase

During the inflammatory phase, activated fibroblasts produce pro-inflammatory cytokines such as TNF-α, interferon-gamma (IFN-γ), IL-6, and IL-12 and release chemokines such as CXCL1, CX3CL1, and CCL2 to recruit immune cells [110,114]. They also interact via ICAM1 and CD40 expression to activate dendritic cells [115], remodel the wound stroma through secretion of matrix metalloproteinases (MMPs), and respond to interstitial flow changes by modifying the microenvironment’s properties [116,117]. Collectively, fibroblasts modulate immune cells’ recruitment, behavior, retention, and survival in damaged tissue, with fibroblast–macrophage crosstalk being particularly important for transitioning from the inflammatory to the proliferation phase, ensuring proper healing progression [107,118].

#### 4.1.4. Influence of Implants’ Surface Properties

In implant-related fibrosis, the interplay among the inflammatory milieu, reactive oxygen species, and the implant’s surface characteristics significantly impact fibroblasts’ behavior and the formation of fibrotic capsules [48,119]. Fibroblasts generate fibrous ECM around implants, rich in Collagen I/III, fibronectin, and proteoglycans [120]. Myofibroblasts, central to this process, form stress fibers and express α-SMA, exerting mechanical forces essential for ECM organization and crosslinking [113,121].

The surface topography of silicone implants significantly influences fibroblasts’ behavior, affecting key processes such as attachment, proliferation, migration, and differentiation. These physical characteristics of the implant modulate fibroblasts’ responses, which may ultimately impact the formation of fibrous capsules [55].

Histological studies identified increased CD3+ T cells and macrophages in capsular biopsies from textured implants, indicating an interplay between T cells and fibroblasts in the fibrotic response [55]. The activation of T cells promotes fibroblast activation and differentiation, contributing to fibrosis. Cytokine profiling of PBMCs’ responses to silicone surfaces revealed matrix-specific differences, especially in IL-6 and TNF-α levels, which influence fibroblast activity. Quantitative RT-PCR analysis showed changes in monocyte/macrophage markers and related cytokines such as TNF-α and IL-1β, further implicating the immune response in fibroblast activation and differentiation [55].

Animal models (rabbits and mice) have demonstrated that implants with smoother surfaces provoke reduced immune responses, characterized by lower levels of inflammatory cytokines and fewer activated fibroblasts [63]. Specifically, implants with an average roughness radius (Ra) of 4 µm exhibit minimal capsular tension lines and rippling, suggesting decreased fibrosis. Conversely, textured implants often show double capsule formation and higher levels of wear debris within capsules, indicating more severe immune reactions and fibrotic encapsulation [63].

Recent clinical studies involving intra- and interindividual analyses in human patients have highlighted the profound influence of the surface microstructure on fibroblasts’ behavior and subsequent fibrotic responses [39,41,59]. Comparisons between SMIs with roughness levels of Ra 60 µm and Ra 4 µm have revealed that reducing surface roughness mitigates early pro-inflammatory responses, while rougher surfaces intensify immune reactions and increase capsular thickness in chronic stages. Rougher surfaces enhance inflammatory signaling pathways such as NF-κB, promoting fibrosis progression, whereas smoother surfaces attenuate inflammation, thereby reducing fibroblast activation and myofibroblast differentiation [30,39,41,55,59,63]. Proteomic analyses indicate that rougher surfaces favor the production of fibrosis-associated proteins, whereas smoother surfaces promote proteins associated with the resolution of fibrosis, thereby influencing ECM remodeling dynamics [30,41,63]. The surface topography thus dictates the initial immune responses and long-term fibrotic outcomes, underscoring its critical role in optimizing implant designs to manage fibrosis [30,39,41,55,59,63].

#### 4.1.5. Sustained Injury and Myofibroblast Differentiation

Under sustained injury or prolonged inflammation, fibroblasts can differentiate into myofibroblasts, contractile cells contributing to tissue contraction and ECM stabilization [122]. This transition is associated with persistent proliferation and resistance to apoptosis, leading to aberrant ECM deposition and tissue dysfunction [123]. Factors such as hypoxia, prevalent near avascular implants, up-regulate HIF-1α, promoting fibrogenesis [124]. Myofibroblasts, stimulated by TGF-β, which is abundant in fibrotic capsules, express α-SMA and other contractile proteins, facilitating wound contraction and ECM remodeling [22,23,24,26]. This process is integral to fibrotic capsule formation, characterized by dense collagen deposition and tissue contraction [125,126,127,128,129].

#### 4.1.6. ECM Remodeling

The ECM environment around silicone implants regulates fibroblast activity and differentiation [28,29,57]. Histological studies reveal alterations in collagen fibers’ orientation and thickness within capsule tissue, indicative of ECM remodeling orchestrated by fibroblasts [130]. Activated fibroblasts remodel the ECM by producing components such as collagen and fibronectin [48,125,128,129,131]. ECM remodeling processes involve proteins such as collagen and MMPs, contributing to the development of capsular fibrosis [80,132,133].

Wound contraction, vascularization decline, ECM turnover, and tensile strength recovery mark the final phase, lasting over a year [134]. Myofibroblasts regulate wound contraction and tissue remodeling, synthesizing ECM proteins and assuming a contractile phenotype [135]. The fibroblast–myofibroblast transdifferentiation is regulated by TGF-β1 and ECM stiffness, with myofibroblasts incorporating α-SMA into stress fibers [136]. Contractile activity plays a crucial role in wound contraction and enhances extracellular matrix (ECM) stiffness, which, in turn, induces the differentiation and persistence of myofibroblasts [137]. The remodeling of the ECM requires a delicate balance between the production of matrix metalloproteinases (MMPs) and ECM proteins. For instance, Collagen III is gradually replaced by Collagen I, leading to an increase in the complexity, organization, and tensile strength of the tissue [138]. Furthermore, apoptosis impacts various cell types, including immune cells, endothelial cells, and myofibroblasts, which reduces vascularization and facilitates the transition from granulation tissue to scar tissue [129]. The presence of silicone implants in the body contributes to the ongoing activation and differentiation of fibroblasts, ultimately resulting in persistent ECM remodeling and the formation of fibrotic tissue [28,29,30].

#### 4.1.7. Implications

Proper timing for inflammation resolution is crucial for successful healing progression, as persistent fibroblast activation and excessive pro-inflammatory mediator production can lead to chronic wounds and fibrosis [20,122]. Excessive fibroblast activity can result in hypertrophic scarring and keloid formation [113,126,139,140,141], while altered signaling pathways, apoptosis failure, and excessive mechanical stress can perpetuate myofibroblast activity [134,136,139]. Myofibroblast dysfunctions can also cause delayed wound healing due to failure in ECM reconstitution [136].

In conclusion, fibroblast activation and differentiation are critical processes in capsular fibrosis associated with silicone implants. Understanding the molecular mechanisms driving these processes, including the influence of surface topography and cytokine signaling, is essential for developing strategies to mitigate fibrotic complications and improve clinical outcomes for patients undergoing implant-based surgeries.

### 4.2. Immune Cell Interactions and Inflammatory Responses

Capsular fibrosis formation around silicone implants involves intricate interactions between biomaterial surfaces and immune cells, influencing inflammation and fibrotic outcomes. Understanding these immune responses at the molecular level is essential for developing targeted therapies and improving clinical outcomes.

#### 4.2.1. Molecular Mechanisms of Immune Cell Activation

Medical devices’ bioperformance and biocompatibility are both directly related to unwanted side effects such as foreign body response, inflammation, and cell adhesion [142]. Immune cells, including macrophages, dendritic cells, and T cells, respond to silicone implantation by recognizing the biomaterial as a foreign body [28]. Macrophages initiate the inflammatory cascade by releasing cytokines such as TNF-α, IL-1β, and IL-6, which stimulate fibroblast activation and collagen deposition around the implant [28,29,41,55,57,63]. Dendritic cells process and present antigens derived from the implant to T cells, initiating adaptive immune responses that are crucial for sustained inflammation and fibrosis [57,63,115].

#### 4.2.2. Role of T Cells in Fibrotic Encapsulation

CD4+ T cells play a pivotal role in regulating immune responses to silicone implants, influencing fibroblasts’ behavior through cytokine secretion and direct cell–cell interactions [57]. Studies have shown increased infiltration of CD4+ T cells in fibrous capsules, correlating with the severity of fibrosis and chronic inflammation [55]. These T cells secrete cytokines such as IFN-γ and TGF-β, which modulate fibroblast activation and promote myofibroblast differentiation [57,63]. The interactions between T cells and other immune cells at the molecular level contribute significantly to the pathogenesis of capsular fibrosis.

Effective healing is usually characterized by a dominant CD4+ T helper 1 (Th1) cell response, whereas a predominant CD4+ T helper 2 (Th2) response and an increase in CD4+ T helper 17 (Th17) cells lead to chronic inflammation, which can ultimately result in fibrosis [29]. Th1 cells mediate tissue damage responses by producing Th1-related pro-inflammatory cytokines (IFN-γ, IL-12) that suppress fibroblast-induced collagen synthesis and attenuate fibrosis [143]. Conversely, Th2 cells mediate adaptive immune responses to injury by producing pro-fibrotic (anti-inflammatory) cytokines (e.g., IL-4, IL-13, IL-10) [143]. As a commonly recognized opponent of Th1 cells, Th2 cells can alter Th1-associated IFN-γ expression levels, and high levels of Th2 cytokines have been reported in several fibrotic diseases [143].

Regulatory T cells (Tregs), another subset of CD4+ T cells, play a critical role in controlling immune responses to self and foreign antigens, thereby preventing autoimmune diseases [144,145,146,147,148,149,150]. Tregs can be divided into two groups. “Natural” Tregs (nTregs), produced by the thymus and characterized by the expression of interleukin-2 receptor (IL-2R) and the forkhead box P3 (Foxp3) transcription factor, exhibit suppressive regulatory activity. Peripheral-induced Tregs (iTregs) arise from the differentiation of naive T cells in the periphery and also secrete anti-inflammatory cytokines such as IL-10 and TGF-β1 [146,148,149,150].

Scarce data exist on specific local side effects (local immune response, activity of immune cells) focusing on lymphocytes isolated from fibrous capsules. The cellular composition of fibrous capsules formed around SMIs was characterized, revealing that macrophages and fibroblasts were the most predominant cell populations in the region abutting the silicone surface (designated as “pseudo-synovium”). Moreover, significant numbers of activated CD4 + CD25 + CD45RO memory T cells were present at this site and adjacent to the vessel [57]. Notably, among the T cells, Treg numbers in peri-SMI fibrotic capsules were inversely proportional to the degree of fibrosis (Baker scores I to IV). Particularly noteworthy was the observation that Tregs were reduced in capsules removed from patients with clinically severe symptoms of capsular contracture (Baker scores III to IV) [57].

Tregs exhibit different transcriptional changes in response to regenerative or fibrogenic environmental cues [151]. The controversy about the role of Tregs in fibrosis is corroborated by the fact that Th17 cells are relatively resistant to Treg suppression [152], and notwithstanding that although TGF-β1 is the most prominent pro-fibrotic cytokine itself, it also induces differentiation of naïve T cells [152].

Tregs isolated from capsules with high-grade fibrosis demonstrated the ability to suppress peripheral T effector cells but exhibited significantly less suppression potential when combined with intracapsular T effector cells [54]. These findings suggest that in the early stages of fibrosis, Tregs play a crucial role in controlling capsular fibrosis by down-regulating Th1/Th17+ effector cells and reducing pro-fibrotic cytokine production.

#### 4.2.3. Influence of Implants’ Surface Properties on T Cell Immune Responses

The physical and chemical properties of silicone implants’ surfaces, such as texture and surface roughness, play a critical role in influencing immune cell adhesion, cytokine secretion, and subsequent fibrotic responses [30,39,41,55,59,63]. Textured surfaces enhance immune cell activation compared with smoother surfaces, exacerbating capsular thickness and fibrotic severity clinically observed with different types of silicone implants [39,41,63].

Research indicates that the chemical composition of implants’ surfaces can significantly influence immune responses. Variations in surface coatings—such as hydrophobic versus hydrophilic—affect protein adsorption and interactions with immune cells, ultimately impacting inflammation and fibrosis. Specifically, hydrophobic surfaces tend to limit protein adsorption due to their lower surface energy. This characteristic restricts the initial protein layer to fewer, more densely packed proteins, potentially limiting the accessibility of binding sites for immune cells and leading to a more restrained immune response. Conversely, hydrophilic surfaces, with higher surface energy, promote greater protein adsorption. This more diverse protein layer enables enhanced immune cell adhesion and activation, which can intensify inflammatory and fibrotic responses around the implant [47,153,154,155,156,157].

In terms of surface topography, metrics such as skewness and kurtosis provide deeper insights beyond simple roughness measurements [93,158]. Surface roughness is characterized by vertical deviations of the actual surface from its ideal form, with larger deviations indicating a rough surface and smaller deviations denoting a smooth surface [93]. Key parameters for assessing surface roughness include the arithmetic mean height (Ra), surface skewness (Ssk), and kurtosis value (Sku), all of which can manipulate friction, cell adhesion, and ultimately tissue response [93,158,159].

Higher skewness values often correlate with sharper peaks, which may enhance the recruitment of inflammatory cells. Conversely, lower kurtosis values might relate to smoother, more biocompatible surfaces [160]. Moreover, an increased surface area from rough surfaces has been linked to enhanced cell adhesion, particularly for fibroblasts, which play a crucial role in tissue response [161].

Research shows that rougher silicone elastomer substrates can decrease fibroblast growth at the sub-micron scale (88–650 nm) [162]. Furthermore, surface topography significantly influences macrophage activation, with rough surfaces up-regulating macrophages’ inflammatory proteins and altering cytokine secretion patterns [163]. This underscores the pivotal role of surface morphology in directing extracellular matrix-related gene expression and the FBR dynamics [164].

A study examining SMIs with patterned surfaces showed that specific matrix metalloproteinases and cytokines were regulated differently, depending on the surface topography. This highlights how these soluble factors orchestrate cellular events such as invasion, blood vessel formation, and fibrosis during the FBR [164].

Thus, understanding the surface topography and its metrics—alongside the chemical composition—can elucidate how implant design influences immune response dynamics, including the development of capsular contracture.

In vitro studies of silicone surfaces co-cultured with human peripheral blood mononuclear cells (PBMCs) revealed that silicone alone did not induce T cell proliferation or significantly modify the distribution of T cell subsets [55]. While silicone does not trigger a significant T cell response, it impacts the surrounding cytokine environment. Differences in silicone’s surface textures resulted in varied cytokine responses, especially regarding IL-6 and TNF-α levels, both of which are critical in inflammation and fibrosis. These findings indicate that the surface texture may affect the severity of inflammatory responses and the resulting fibrotic encapsulation. Textured implants tend to provoke more pronounced immune responses than smooth variants, with these surfaces associated with heightened cytokine secretion, such as IL-6 and TNF-α, which may lead to increased rates of capsular contracture.

Adhesion and gene expression analyses demonstrated that textured surfaces could alter macrophages’ behavior, as evidenced by changes in markers such as CD14, CD68, and others [55]. This interaction is pivotal for elucidating the underlying mechanisms of capsular contracture and fibrosis.

In vivo studies in patients examined the impact of reducing implants’ surface roughness from Ra 60 µm to Ra 4 µm on inflammatory tissue repair following implantation [41]. Flow cytometric analysis revealed no significant difference in the distribution of CD4+ T cell subpopulations (including TH1, TH17, CM, and EM) between smoother (Ra 4 µm) and rougher (Ra 60 µm) SMI surfaces, indicating that surface topography did not directly influence the proliferation or distribution of these T cell subsets around implants. Despite this, cytokine secretion analysis showed a pronounced TH1 response characterized by increased secretion of IFN-γ, IL-1b, and TNF-α around both SMI types, suggesting robust TH1-mediated responses irrespective of the surface roughness. Gene expression analysis indicated elevated levels of IFN-γ and IL-17 around rougher surfaces (Ra 60 µm) compared with smoother surfaces (Ra 4 µm), indicating a heightened pro-inflammatory and pro-fibrotic T cell response around rougher implants. Correlation analysis further supported these findings, showing significant positive correlations between IL-17A secretion and TH17 cells, as well as between IFN-γ expression and TH1 cells, specifically in wounds enclosed by 60 µm SMIs, highlighting the interplay between T cells’ cytokine production and immune cell profiles influenced by implants’ surface roughness [41].

The study found a consistent macrophage response around both SMI types, indicating no significant difference in macrophages’ presence or distribution based on surface roughness. However, gene expression analysis revealed differences in macrophage polarization between the surfaces. Notably, fibrotic tissue surrounding the rougher implant exhibited increased expression of M1 markers, such as IFN-γ and CCL2, which are indicative of a pro-inflammatory macrophage phenotype. In contrast, the capsule enclosing the smoother implant showed elevated levels of M2 markers, including IL-4 and IL-10, suggesting a shift toward an anti-inflammatory or pro-tissue repair phenotype. Additionally, immunohistological analysis of the intracapsular environment demonstrated enrichment of CD25+ and Foxp3+ immune cells around the rougher silicone mammary implant, which are associated with Treg cells [41]. This finding implies a regulatory role for these cells in dampening immune responses and potentially modulating tissue repair processes.

#### 4.2.4. Macrophages in the Context of SMIs: Cellular Interactions and Inflammatory Responses

Macrophages play a pivotal role as early responders to surgically placed implants, accumulating at the implantation site for extended periods to phagocytose cellular debris and implant abrasion products [165]. Their presence triggers the up-regulation of pro-inflammatory and pro-fibrotic cytokines such as IL-1, IL-8, MCP-1, CXCL13, and MIP, which further recruit macrophages and modulate their activity during the FBR [166,167,168]. Despite their efforts, macrophages often fail to completely engulf large implants, leading to frustrated phagocytosis and chronic inflammation [169]. This chronic inflammatory state contributes to the formation of foreign body giant cells (FBGC) and exerts trophic actions on vascular cells, adaptive immune cells, and fibroblasts, ultimately contributing to implant fibrosis [170,171,172].

Studies using animal models have demonstrated that abolishing macrophage recruitment through methods such as clodronate liposome-induced depletion can significantly reduce monocyte infiltration, FBGC formation, neovascularization, and fibrosis around implants [165,170,173]. Furthermore, targeting macrophage receptors such as colony-stimulating factor-1 receptor (CSF-1R), which is up-regulated post-implantation, has shown promising results in suppressing implant fibrosis [165].

#### 4.2.5. Strategies Targeting Macrophages Through Implant Surface Modifications

The objectives of implant surface modifications targeting macrophages are twofold: (1) to suppress the formation of detrimental macrophage phenotypes while stimulating regenerative and resolving activation states [174,175,176] and (2) to prevent the formation of FBGCs, which typically follow the chronic inflammatory phase of the FBR [171].

Macrophages exhibit diverse activation states, broadly categorized into pro-inflammatory M1 and pro-regenerative (yet pro-fibrotic) M2 types [175,176]. M1 macrophages dominate the early phases of the FBR, producing cytokines such as PDGF, TNF-α, IL-6, G-CSF, and GM-CSF, which are crucial for inflammation and tissue remodeling [166,177]. In contrast, M2 macrophages, induced by cytokines such as IL-4 and IL-13, play roles in tissue repair but can also promote fibrosis and FBGC formation [178].

The surface chemistry and topography of implants significantly influence macrophages’ responses, including recruitment, adhesion, spreading, and activation [179]. For instance, macrophages attach to implant surfaces via integrins and sense surface characteristics through Toll-like receptors and scavenger receptors [180,181,182]. Modulating these interactions alters macrophages’ attachment, spreading, and fusion into FBGCs [178,183,184].

Studies have highlighted that micro-roughness features within a specific range (e.g., 0.51–1.36 μm) promote M2-like phenotypes in macrophages, influencing their cytokine profiles and fusion into FBGCs [185]. Similarly, nano-patterned surfaces with defined features influence macrophages’ behaviors, affecting their inflammatory profiles and phagocytic activities [186,187,188]. Additionally, the substrate’s stiffness plays a critical role, with stiffer materials promoting pro-inflammatory responses and FBGC formation in macrophages compared with softer substrates [189,190].

Reducing the implants’ surface roughness from Ra 60 µm to Ra 4 µm in vivo in human patients revealed the consistent presence of macrophages around both types of surfaces. Gene expression analysis uncovered distinct macrophage polarization patterns, with capsular tissue surrounding the rougher implants exhibiting heightened expression of M1 markers such as IFN-γ and CCL2. In contrast, capsules enveloping the smoother implants showed increased expression of M2 markers such as IL-4 and IL-10. The immunohistological analysis further indicated an enrichment of CD25+ and Foxp3+ immune cells around rougher surface implants, suggesting a role for T regulatory cells in attenuating immune responses and influencing tissue repair processes.

Understanding macrophages’ behaviors and responses to implant surfaces is crucial for developing strategies to mitigate implant fibrosis and enhance biocompatibility. Targeted modifications of surface properties can alter macrophages’ activation states, influencing their inflammatory profiles and their propensity to form foreign body giant cells (FBGCs). These modifications may promote an anti-fibrotic macrophage phenotype and modulate T cell responses, potentially reducing the incidence and severity of fibrotic encapsulation.

### 4.3. Silicone Gel Bleed and Its Impact on Immune Response and Fibrosis

Silicone gel bleed refers to the migration of low molecular weight silicone compounds through the elastomeric envelope of breast implants, initiating significant biological effects in the surrounding tissues [191]. This phenomenon leads to a cascade of immunological responses that can culminate in capsular fibrosis and other complications [192,193].

Research indicates that silicone particles provoke a chronic inflammatory response in the periprosthetic tissue, correlating the presence of silicone in the capsule to an increased incidence of Baker Grade IV capsular contracture [191]. The accumulation of silicone materials in capsular tissue emphasizes how silicone gel bleed can provoke an inflammatory response, leading to significant pathological changes in the surrounding tissue. This sustained immune reaction results from interactions between silicone particles and immune cells, particularly T cells, which can activate and form granulomas—aggregates of macrophages attempting to isolate foreign materials [191,194,195]. Notably, the inflammatory pathways activated by silicone gel bleed may parallel those seen with orthopedic implants, where debris from implants also triggers significant immune responses, leading to chronic inflammation and tissue remodeling [196]. The chronic inflammation caused by silicone gel bleed can lead to the formation of granulomas, which are collections of macrophages that attempt to isolate the foreign material [194,195]. These granulomas can become fibrotic, further contributing to capsular contracture. Understanding the specific pathways through which silicone induces granuloma formation could help identify potential therapeutic targets to reduce fibrosis. This inflammatory milieu contributes to fibrosis and capsule thickening over time.

In addition to their role in capsular contracture, granulomas formed in response to silicone particles may also play a critical role in the potential oncogenic effects of silicone, as the inflammatory environment they create can trigger metaplastic changes that predispose breast tissue to malignancy. Moreover, silicone gel bleeding may facilitate tumor oncogenesis within breast tissue by triggering metaplastic changes in response to injury, further emphasizing its potential long-term repercussions on breast health [193,197]. This suggests that the impact of silicone extends beyond capsular contracture to potentially influence breast tissue carcinogenesis, particularly through inflammatory pathways. Interestingly, similar chronic inflammation has been documented in orthopedic implants, where particulate debris can lead to systemic health issues and delayed hypersensitivity reactions, underscoring the need for more comprehensive studies of breast implant debris [198]. Furthermore, emerging studies suggest a significant association between silicone implants and autoimmune diseases [199,200], highlighting the need for further investigation into how chronic inflammation from silicone exposure may contribute to these systemic health issues. Some studies suggest a correlation between silicone implants and autoimmune diseases, possibly due to the continuous inflammatory state initiated by silicone particles [191]. Patients with silicone implants have reported symptoms consistent with autoimmune disorders, raising questions about whether silicone gel bleed contributes to these systemic effects through a sustained immune response.

Silicone leakage can trigger regional immune responses, leading to lymphadenopathy that complicates the diagnosis of breast pathologies. This situation underscores the challenges associated with silicone implants, as the immune reaction may obscure the differentiation between benign and malignant conditions. Additionally, the sustained immune activation resulting from silicone exposure may have systemic implications, potentially resulting in further complications in patient evaluation and management [191,192]. This connection highlights the broader implications of chronic inflammation induced by silicone, as sustained immune activation may predispose patients to hematological malignancies [191]. The presence of silicone in the pericapsular tissue has been linked to not only local inflammatory responses but also systemic health implications. For instance, persistent silicone exposure could lead to changes in lymphatic function and potentially increase the risk of regional lymphadenopathy [192,197]. Additionally, the implications for breast cancer risk due to chronic inflammation and metaplastic changes warrant further exploration [197].

Granulomas formed in response to silicone particles can lead to fibrotic changes, further complicating the tissue environment and contributing to capsular contracture. The relationship between silicone exposure and potential autoimmune reactions has raised concerns about the long-term health risks associated with silicone implants. In orthopedic literature, implant debris is known to provoke both innate and adaptive immune responses, a phenomenon that may also occur with silicone gel bleed, indicating a need for further research on the long-term implications of immune activation in breast tissue [196]. Understanding the molecular mechanisms behind capsular contracture and the role of pro-fibrotic cytokines in fibroblast activation may lead to targeted therapies to mitigate these complications [195].

The advancements in imaging techniques not only enhance our understanding of silicone’s distribution but also hold the promise of significantly improving patient outcomes by enabling more effective monitoring of implants’ integrity and timely interventions for any emerging complications. Innovative imaging techniques significantly enhance the understanding of silicone’s distribution within tissues and its potential roles in pathophysiology. Advances in imaging techniques, such as stimulated Raman scattering, enhance the detection of silicone materials in tissues, allowing for better assessment of gel bleed and its impact on the surrounding structures [201]. Improved imaging modalities may aid in the early diagnosis of complications related to silicone implants, facilitating timely interventions.

As research advances, these findings will be vital for developing strategies to mitigate the adverse effects of silicone implants, ensuring better patient outcomes and a deeper understanding of the complex interplay between implant materials and host immune responses.

### 4.4. Implications for Clinical Practice

Cellular and tissue-level studies have elucidated critical insights into the mechanisms underlying capsular fibrosis associated with SMIs. Key findings (Table 1) underscore the pivotal roles of fibroblasts and immune cells, particularly macrophages and T cells, and the influence of the implants’ surface properties in driving inflammatory and fibrotic responses.

Future research should continue to explore how specific surface modifications can effectively enhance biocompatibility while balancing reduced inflammatory responses, thereby improving the longevity and safety of SMIs.

In conclusion, comprehensive insights into fibroblasts’ behavior, immune cells’ interactions, and the impact of implants’ surface properties provide a foundation for advancing therapeutic approaches aimed at minimizing complications associated with SMIs. Continued research efforts are essential to refine our understanding and translate these findings into clinically effective strategies for enhancing patient outcomes in implant-based surgery.

## 5. Microbial Interactions and Biofilm Formation on SMIs: Implications for Capsular Contracture

### 5.1. The Race for the Surface: Host Cells vs. Bacteria

Upon the implantation of a breast implant, a “race for the surface” occurs, in which host cells (such as macrophages, fibroblasts, and platelets) compete with bacteria to occupy the implant’s surface [202]. Although implanted under specific sterilization and disinfection guidelines [203,204,205], microbial colonization and biofilm formation on the implant’s surface can occur. Biofilms are communities of bacteria encased in a protective matrix, making them resistant to the immune response and antibiotics [206]. The antimicrobial immune response may be activated as a part of the overall immune reaction [29]. These biofilms act as bacterial reservoirs and are sources of chronic and/or subclinical infections [207].

As bacteria approach the implant, they encounter Van der Waals, electrostatic, and hydrophobic interactions with the implant’s surface, leading to initial adherence [153,208]. This is followed by more permanent site-specific interactions, where bacterial pili and fimbriae form attachments with the biomaterial’s surface or its conditioning film [209]. At this point, bacteria transition from their planktonic to sessile state. Biofilm formation proceeds with rapid bacterial proliferation, production of extracellular polymeric substances (EPSs), and a phenotypic shift contributing to the resilient nature of the biofilm’s growth [210]. Within the biofilm, bacteria communicate via quorum sensing, releasing signaling molecules to coordinate gene expression [211]. Biofilm bacteria can also detach and revert to their planktonic state, dispersing to colonize new surfaces [212]. In the context of SMIs, this could involve other areas on the implant or different tissues and regions of the body.

Many bacterial species produce binding proteins specific to collagen and fibronectin, which form the fibrous capsule around the breast implant [213,214]. This facilitates bacterial attachment and adherence to the capsule, followed by proliferation and biofilm formation. Increased numbers of fibroblasts correlate with a higher incidence of capsular contracture, and more bacteria are found on contracted capsules compared with non-contracted ones [215]. A porcine model by Tamboto et al. demonstrated that biofilm formation on and around SMIs is associated with a fourfold increased risk of developing capsular contracture [216].

The most common bacterial species detected on SMIs are Staphylococcus spp., particularly *Staphylococcus epidermidis* [217]. These species are normal skin flora, suggesting contamination of the implant or site during surgery. In a rat model, Miller et al. reported that the hematogenous spread of *Staphylococcus aureus* from a remote infection increased capsule thickness, myofibroblast numbers, and collagen density around implanted silicone blocks [218].

### 5.2. Biofilm Formation and Its Implications for SMIs

In a recent study, moving to an in vitro setting, the evaluation of SMIs’ surface patches with diverse topographies revealed significant effects on microbial adhesion, growth, and colonization by *S. epidermidis* and *S. aureus* [59]. Both bacterial species adhered to and colonized all tested surfaces, but the presence of silicone markedly inhibited their growth and colonization compared with controls without silicone patches [59]. The topography of the silicone patch significantly influenced bacterial growth, with increased texture correlating positively with enhanced bacterial colonization. Biofilm formation, a recognized virulence factor in biomaterial-related infections, was evident on textured surfaces after inoculation with *S. epidermidis* or *S. aureus*. The topography of the silicone patch significantly influenced bacterial growth and colonization, with enhanced texture correlating with increased bacterial growth [59].

Antimicrobial immune responses refer to the body’s defense mechanisms against microbial invaders, such as bacteria. In the context of SMIs, the immune response is typically triggered by the presence of foreign material (silicone) in the body, which involves the activation of immune cells and processes aimed at removing or isolating the foreign material [166,219,220]. The initial response to SMIs includes an inflammatory reaction. Inflammation is a part of the immune response and involves the recruitment of immune cells to the implant site to clear debris and potentially harmful substances [29]. While silicone itself is not a microbial agent, the immune system may respond to the implant by releasing antimicrobial substances as a general defense mechanism.

A diverse array of proteins linked to immune response, inflammation, and wound healing was discovered in the vicinity of SMIs [30]. In vivo analysis of SMIs’ surface-associated proteome, including plasma, local tissue, and early fibrosis stages, revealed a significant inflammatory storm within the first five days post-implantation, with antimicrobial agents adhering to the SMIs’ surface over the next 6 to 8 months [30,41]. Notably, 65 plasma-derived components were involved in the antimicrobial humoral response, with FLG2 exclusively associated with rougher SMI surfaces, indicating a chronic antimicrobial inflammatory response [59]. Next-generation sequencing (NGS) analysis of wound-associated microbiomes revealed significant topography-specific variations, with higher microbial diversity and quantity on rougher surfaces [59]. A skin microbiome assessment at the incision sites identified only eight species, with *Staphylococcus hominis* and *Staphylococcus epidermidis* being prevalent. This finding indicates that the types of bacteria present are related to the surgical environments where they were found. Staphylococci, identified in acute and chronic wounds and on SMIs’ surfaces, emphasize the need to understand individual skin microbiomes in surgical contexts [59].

### 5.3. Antimicrobial Immune Responses and Proteomic Insights in Capsular Fibrosis

**Figure 4:**
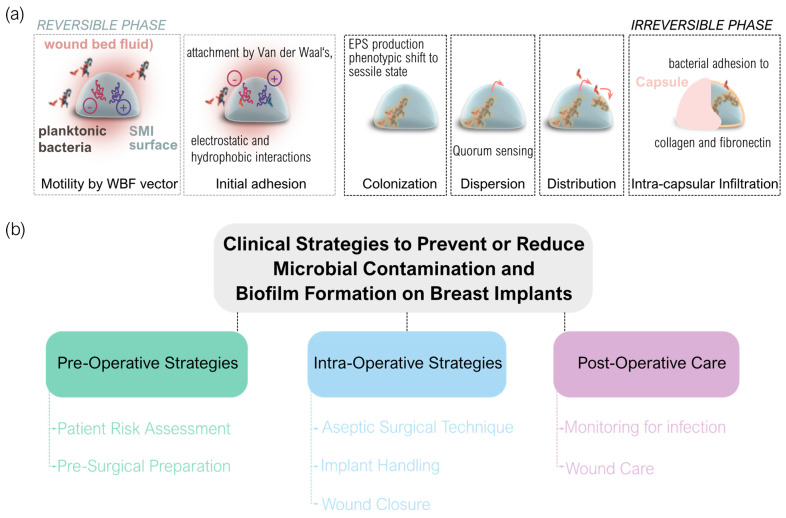
Bacterial adhesion, biofilm formation on SMIs’ surfaces, and clinical strategies for their prevention. Bacterial colonization of SMIs’ surfaces and the subsequent formation of biofilms are major contributors to chronic infection and the development of complications such as capsular contracture. Various clinical strategies are employed to minimize microbial contamination and biofilm formation at different stages of the surgical process. (**a**) Bacterial adhesion to the SMI’s surface, colonization, and biofilm formation. *Reversible phase:* Planktonic bacteria initially adhere to the SMI’s surface through electrostatic and hydrophobic interactions. This phase is reversible, and bacteria can be removed if appropriate interventions are applied. Irreversible phase: Over time, bacteria firmly attach to the SMI’s surface, proliferate, and form biofilms. The biofilm matrix protects bacteria from the immune response and antimicrobial agents, leading to persistent infection. Capsule formation: The presence of bacterial biofilms and the continuous inflammatory response contribute to the formation and thickening of the fibrous capsule, leading to capsular contracture. (**b**) Flowchart illustrating the clinical strategies for preventing or reducing microbial contamination and biofilm formation on SMIs. Pre-operative strategies focus on assessing patient risk, preparing the surgical site through sterilization and disinfection, and choosing implants with surface modifications to reduce bacterial adhesion. Intra-operative practices emphasize maintaining a sterile environment, proper handling of the implant, and using antibiotic interventions to minimize contamination. Post-operative care includes monitoring the antimicrobial inflammatory response, administering antibiotics, and maintaining meticulous wound care to detect and prevent early signs of infection.

To gain deeper insight into the mechanisms driving capsular fibrosis around SMIs, it is crucial to consider the significant role of antimicrobial strategies and inflammatory responses (Figure 4). Key processes such as sterilization, disinfection, and surface modifications (Figure 4a) of SMIs are essential in minimizing microbial contamination. Both immediate and chronic antimicrobial inflammatory responses play a role in this context, with persistent microbial contamination leading to ongoing inflammation and fibrous capsule formation (Figure 4b). Furthermore, bacterial adhesion and biofilm formation are critical stages that influence the onset of capsular contracture (Figure 4). These considerations underscore how antimicrobial interventions and inflammatory dynamics are integral to the development of capsular fibrosis and highlight potential strategies for mitigating these challenges.

## 6. Clinical Translation and Future Directions

Advances in biomaterial science and molecular studies have illuminated new pathways for enhancing clinical outcomes in breast implant surgery.

One of the significant potentials lies in personalized risk assessment and management strategies. By deciphering individual variations in immune responses and microbial colonization patterns, clinicians can tailor surgical approaches to mitigate risks such as capsular contracture. This personalized approach may involve optimized implant selection and targeted antimicrobial prophylaxis. Such strategies aim to improve patient outcomes by reducing complications associated with immune reactions and biofilm formation.

Furthermore, the development of biomaterials and implant modifications stands at the forefront of innovation. Insights from molecular studies and preclinical models are driving the creation of next-generation implants designed to minimize biofilm formation and inflammatory responses. These advancements include the exploration of anti-biofilm coatings, immunomodulatory surfaces, and materials that mimic natural tissue interactions. By improving implants’ biocompatibility and longevity, these innovations hold the potential to enhance patient satisfaction and reduce the need for revision surgeries.

In parallel, the identification of novel therapeutic targets through molecular pathways offers promising avenues for targeted interventions. Potential therapies such as anti-inflammatory agents, quorum-sensing inhibitors, and biofilm disruptors are being investigated to prevent or mitigate fibrotic reactions post-implantation. These targeted treatments aim to modulate immune responses and improve the overall biointegration of implants, thereby advancing the field towards more effective management of complications and improved long-term patient outcomes.

Moreover, advancements in surgical techniques and post-operative care protocols are being informed by molecular insights. Understanding the intricate mechanisms of wound healing and immune modulation guides refinements in surgical approaches. This includes strategies to minimize tissue trauma during implantation, optimize tissue integration around implants, and enhance infection prevention measures. By implementing evidence-based practices derived from molecular and preclinical research, clinicians can strive to optimize surgical outcomes and reduce the incidence of complications.

Long-term monitoring and patient education are also pivotal aspects influenced by molecular studies. Comprehensive monitoring protocols informed by molecular biomarkers [30] can facilitate the early detection of complications and prompt intervention. Equally important is patient education, which empowers individuals with knowledge about their implants and potential risks. Educating patients on the importance of regular follow-up evaluations and self-monitoring can promote proactive management and early intervention, contributing to improved long-term implant success and patient satisfaction.

Lastly, collaborative research efforts and rigorous clinical trials are essential for translating scientific discoveries into clinical practice. Interdisciplinary collaboration among researchers, clinicians, and industry stakeholders accelerates the validation of emerging technologies and therapies. This collaborative approach ensures that advancements in breast implant surgery are evidence-based and patient-centered, fostering continuous improvement and innovation in the field of plastic and reconstructive surgery.

In conclusion, the clinical translation of insights from molecular studies and preclinical models holds immense promise for revolutionizing breast implant surgery. By integrating these advancements into clinical practice, clinicians can strive to enhance implant biocompatibility, reduce complications, and improve overall patient outcomes. Continued research, interdisciplinary collaboration, and evidence-based practice are crucial for realizing these goals and advancing the standard of care in breast implant surgery.

## 7. Challenges and Limitations

Despite significant advancements, several challenges and limitations persist in the current research on breast implant surgery, particularly concerning immune responses and novel interventions.

One of the primary challenges is the variability in individual immune responses to implants. While molecular studies have elucidated key pathways involved in immune reactions, there remains substantial variation among patients in how their immune systems react to implanted materials. This variability can influence the risk of complications such as capsular contracture and infection, making it challenging to predict outcomes and tailor personalized treatment strategies effectively.

Another unresolved question pertains to the long-term outcomes of novel interventions aimed at mitigating complications associated with SMIs. While preclinical models and early clinical studies have shown promise for therapies targeting biofilm formation, inflammation, and implant integration, the durability and effectiveness of these interventions over extended periods remain uncertain. Longitudinal studies are needed to assess the sustainability of the therapeutic benefits and potential long-term risks associated with new biomaterials and treatments.

Furthermore, the complexity of biofilm-related infections poses a significant challenge in breast implant surgery. Biofilms are resilient communities of bacteria encased in an extracellular matrix, which makes them resistant to immune responses and conventional antibiotics. Despite advances in understanding biofilms’ formation and their impact on capsular contracture, effective strategies to prevent and eradicate biofilms on implants remain elusive. Future research efforts should focus on developing innovative anti-biofilm strategies that can be translated into clinical practice.

The translational gap between benchtop research and clinical implementation presents a persistent limitation. While preclinical models provide valuable insights into biological mechanisms and therapeutic targets, translating these findings into safe and effective clinical interventions requires rigorous validation in human studies. Bridging this gap necessitates collaborative efforts among researchers, clinicians, and regulatory bodies to ensure that novel interventions meet stringent safety and efficacy standards before widespread adoption.

Furthermore, the influence of silicone curing techniques, such as platinum and tin curing, on immune responses and capsular fibrosis remains an important area for further investigation. Currently, evidence directly linking these curing methods to immune responses is limited. Future research is necessary to elucidate their effects on macrophages’ behavior and the foreign body response. Addressing this knowledge gap will be critical as advancements in silicone formulations and curing processes continue to evolve in the field of breast implants.

Ethical considerations also pose challenges in breast implant research, particularly regarding patient consent, long-term surveillance, and communication of the risks and benefits associated with implants. Ensuring comprehensive informed consent and empowering patients with accurate information about the potential risks, including immune responses and long-term outcomes, is essential for promoting patient autonomy and shared decision-making in surgical settings.

In conclusion, while molecular insights and preclinical breakthroughs have significantly advanced our understanding of breast implant surgery, several challenges and unanswered questions remain (Table 2). Addressing variability in immune responses, assessing the durability of novel interventions, combating biofilm-related infections, closing the translational gap, and navigating ethical considerations are critical priorities for future research. By addressing these challenges collaboratively and systematically, researchers and clinicians can strive to optimize patient outcomes and advance the field toward safer, more effective breast implant surgery practices.

## 8. Conclusions

In conclusion, the exploration of immune mechanisms underlying SMI-associated capsular fibrosis represents a crucial frontier in aesthetic and reconstructive surgery. Through this review, we have delved into the intricate interplay of host responses and microbial interactions, highlighting both the progress made and the challenges that lie ahead.

The findings underscore the multifaceted nature of immune responses to SMIs, shaped by factors ranging from the biomaterial’s properties to individual variations in immune profiles. Despite these complexities, advancements in molecular insights and preclinical models offer promising avenues for improving clinical outcomes. By elucidating the key pathways involved in capsular fibrosis, such as inflammation, biofilm formation, and tissue integration, researchers are paving the way for targeted interventions that could mitigate complications and enhance the longevity of implants.

Looking forward, ongoing research holds the potential to catalyze transformative breakthroughs in treatment strategies for breast implant patients. Future studies focusing on personalized medicine approaches, innovative biomaterials, and novel antibiofilm therapies are poised to redefine the standards of care in plastic surgery. Moreover, the integration of patient-centric outcomes, ethical considerations, and regulatory frameworks will be pivotal in translating research findings into safe and effective clinical practices.

In essence, the journey toward unraveling the immune web of SMI-associated capsular fibrosis is not just about understanding biological mechanisms; it is about improving quality of life. By harnessing the collective efforts of scientists, clinicians, and patients, we can envisage a future where complications are minimized, outcomes are optimized, and patient care in plastic and reconstructive surgery reaches new heights of excellence. As we continue to push the boundaries of knowledge and innovation, the promise of achieving these goals remains within our grasp.

## Figures and Tables

**Table 1:** Key cellular and immune mechanisms in fibrosis around SMIs.

Cellular/Immune Mechanism	Role in Fibrosis and Implant Interaction	Molecular Mechanisms and Key Markers	Clinical Implications
Fibroblast activation	Fibroblasts respond to the presence of silicone implants by activating into myofibroblasts, key cells in wound healing and fibrosis processes.	TGF-β1, α-SMA, and SMAD proteins. TGF-β1 signaling leads to SMAD protein activation, promoting α-SMA expression and fibroblast activation.	Persistent activation can lead to excessive fibrosis, contributing to complications such as capsular contracture.
Myofibroblast differentiation	Prolonged inflammation and mechanical stress trigger fibroblasts to differentiate into myofibroblasts, producing ECM components and driving tissue contraction.	TGF-β1, α-SMA, and Collagen I/III. TGF-β1 induces α-SMA and collagen production, facilitating myofibroblast differentiation and ECM contraction.	Myofibroblast activity is a key driver of fibrotic capsule formation around implants, impacting implant outcomes.
ECM remodeling	Fibroblasts and MMPs continuously remodel the ECM around silicone implants, balancing collagen deposition and degradation.	MMPs, TIMPs, and Collagen I/III. MMPs degrade ECM components, while TIMPs inhibit MMPs to regulate ECM remodeling.	Dysregulated ECM remodeling can result in a stiffer, thicker fibrotic capsule, complicating implant removal or revision surgery.
Immune cell activation	Immune cells, including macrophages, dendritic cells, and T cells, are activated by silicone implants, releasing cytokines that further stimulate fibroblasts.	IL-1β, TNF-α, IL-6, and TGF-β. Pro-inflammatory cytokines such as IL-1β and TNF-α activate fibroblasts and sustain chronic inflammation.	Chronic immune activation can perpetuate fibrosis and contribute to implant-related complications such as chronic inflammation.
Macrophage polarization	Macrophages polarize into M1 (pro-inflammatory) or M2 (anti-inflammatory) phenotypes in response to the implant’s properties, affecting fibrosis.	CD86 (M1), CD206 (M2), IL-10, and TGF-β. M1 macrophages express CD86 and produce IL-1β, while M2 macrophages express CD206 and secrete IL-10 and TGF-β.	Targeting macrophage polarization through surface modifications could reduce fibrotic responses and improve implants’ biocompatibility.
T cell-mediated responses	CD4+ T cells, particularly the Th1 and Th2 subsets, regulate fibrosis, with Th1 promoting and Th2 potentially reducing fibrotic responses.	IFN-γ (Th1), IL-4, and IL-13 (Th2). Th1 cells produce IFN-γ, driving fibrosis, while Th2 cells secrete IL-4 and IL-13, which may reduce fibrosis.	Targeting T cell responses through specific immunomodulatory therapies, such as the use of monoclonal antibodies to block Th1 cytokines (e.g., IFN-γ) or promoting Th2 responses with IL-4 or IL-13, could help mitigate fibrosis around silicone implants.
Regulatory T cells (Tregs)	Tregs modulate the immune response by suppressing excessive immune activation and maintaining immune tolerance around implants.	FOXP3, IL-10, and TGF-β. FOXP3 is a key marker for Tregs, which secrete IL-10 and TGF-β to suppress inflammation and fibrosis.	Enhancing Tregs’ activity could help in reducing chronic inflammation and fibrosis, improving implants’ biocompatibility.

**Table 2:** Clinical translation and future directions in SMI-based breast surgery.

Focus Area	Advancements	Clinical Translation	Future Directions
Personalized risk management	- Tailored patient care by analyzing immune responses and microbial patterns (risk mitigation for capsular contracture).	- Personalized implant selection, antimicrobial prophylaxis, and post-operative plans to reduce complications.	- Further studies to better understand patient-specific immune responses to enable more precise and effective personalized care.
Biomaterials and implant modifications	- Development of advanced materials to reduce biofilm formation and inflammation (e.g., anti-biofilm coatings, immunomodulatory surfaces).	- New biomaterials designed for biocompatibility, longevity, and lower complication rates, potentially reducing the need for revision surgeries.	- Continued innovation in materials mimicking natural tissues and enhancing implant integration for better biocompatibility and reduced immune reactions.
Targeted therapeutic approaches	- Exploration of therapies such as anti-inflammatory agents, quorum-sensing inhibitors, and biofilm disruptors to prevent fibrotic reactions.	- Modulation of immune responses to improve biointegration and reduce fibrotic complications post-implantation.	- Identifying and testing new therapeutic targets through molecular studies to prevent or mitigate complications.
Surgical techniques and post-operative care	- Improved techniques minimizing tissue trauma, optimizing tissue integration, and preventing infections through molecular insights.	- Implementation of evidence-based surgical practices to enhance precision, minimize trauma, and improve recovery outcomes.	- Further refinement of techniques based on molecular and preclinical data to reduce complications and improve healing.
Patient monitoring and Educatione	- Use of molecular biomarkers for the early detection of complications; an emphasis on patient education regarding self-monitoring and follow-up care.	- Development of monitoring protocols for early intervention, along with improved patient engagement for long-term implant success.	
Personalized medicine	Generalized treatments may not consider individual patient factors, leading to suboptimal outcomes.	Uniform antibiotic and anti-fibrotic regimens based on general risk profiles.	Personalized treatments tailored to patient-specific factors (microbial flora, immune responses, genetic predisposition).

## Data Availability

No new data were created or analyzed in this study. Data sharing is not applicable to this article.

## References

[1] Hasin Y., Seldin M., Lusis A. (2017). Multi-omics approaches to disease. Genome Biol..

[2] Barh D., Zambare V., Azevedo V. (2013). Omics: Applications in Biomedical, Agricultural, and Environmental Sciences.

[3] Aderem A. (2005). Systems biology: Its practice and challenges. Cell.

[4] Yan S., Nagle D.G., Zhou Y., Zhang W. (2018). Application of Systems Biology in the Research of TCM Formulae. Systems Biology and Its Application in TCM Formulas Research.

[5] Miho E., Yermanos A., Weber C.R., Berger C.T., Reddy S.T., Greiff V. (2018). Computational Strategies for dissecting the high-dimensional complexity of adaptive immune repertoires. Front. Immunol..

[6] Schussek S., Trieu A., Doolan D.L. (2014). Genome- and proteome-wide screening strategies for antigen discovery and immunogen design. Biotechnol. Adv..

[7] Singh S., Sarma D.K., Verma V., Nagpal R. (2023). Unveiling the future of metabolic medicine: Omics technologies driving personalized solutions for precision treatment of metabolic disorders. Biochem. Biophys. Res. Commun..

[8] Chavda V., Bezbaruah R., Valu D., Desai S., Chauhan N., Marwadi S., Deka G., Ding Z. (2023). Clinical Applications of “Omics” Technology as a Bioinformatic Tool. Bioinformatics Tools for Pharmaceutical Drug Product Development.

[9] Quiroz I.V. Exploring the Intersection of Omics Technologies and Biotechnology in Drug Interaction Studies. In *Biotechnology and Drug Development*; 2024; p. 188. https://books.google.com/books?hl=de&lr=&id=EEb9EAAAQBAJ&oi=fnd&pg=PA188&dq=application+of+omics+technologies+in+biomedical+and+pharmaceutical+research.&ots=u6sMBjDv5b&sig=H6rlgX4u3iryxa8SXaFFJAlzPtk.

[10] Stein C.M., Weiskirchen R., Damm F., Strzelecka P.M. (2021). Single-cell omics: Overview, analysis, and application in biomedical science. J. Cell Biochem..

[11] Aga A.M., Woldesemayat A.A. (2024). Application of Omics and Bioinformatics Technologies in Response to COVID-19 Pandemic. Mol. Cell. Biomed. Sci..

[12] Bai J.P.F., Melas I.N., Hur J., Guo E. (2018). Advances in omics for informed pharmaceutical research and development in the era of systems medicine. Expert Opin. Drug Discov..

[13] Dai X., Shen L. (2022). Advances and Trends in Omics Technology Development. Front. Med..

[14] Davis M.M., Tato C.M., Furman D. (2017). Systems immunology: Just getting started. Nat. Immunol..

[15] Bonaguro L., Schulte-Schrepping J., Ulas T., Aschenbrenner A.C., Beyer M., Schultze J.L. (2022). A guide to systems-level immunomics. Nat. Immunol..

[16] Pulendran B., Davis M.M. (2020). The science and medicine of human immunology. Science.

[17] Devenish L.P., Mhlanga M.M., Negishi Y. (2021). Immune Regulation in Time and Space: The Role of Local- and Long-Range Genomic Interactions in Regulating Immune Responses. Front. Immunol..

[18] Wynn T.A. (2007). Common and unique mechanisms regulate fibrosis in various fibroproliferative diseases. J. Clin. Investig..

[19] Ji L., Wang T., Tian L., Song H., Gao M. (2020). Roxatidine inhibits fibrosis by inhibiting NF κB and MAPK signaling in macrophages sensing breast implant surface materials. Mol. Med. Rep..

[20] Wynn T.A. (2008). Cellular and molecular mechanisms of fibrosis. J. Pathol..

[21] Kuo Y.L., Jou I.M., Jeng S.F., Chu C.H., Huang J.S., Hsu T.I., Chang L.R., Huang P.W., Chen J.A., Chou T.M. (2019). Hypoxia-induced epithelial-mesenchymal transition and fibrosis for the development of breast capsular contracture. Sci. Rep..

[22] Biernacka A., Dobaczewski M., Frangogiannis N.G. (2011). TGF-β signaling in fibrosis. Growth Factors.

[23] Chaikuad A., Bullock A.N. (2016). Structural basis of intracellular TGF-β signaling: Receptors and smads. Cold Spring Harb. Perspect. Biol..

[24] Meng X.M., Nikolic-Paterson D.J., Lan H.Y. (2016). TGF-β: The master regulator of fibrosis. Nat. Rev. Nephrol..

[25] Lan H.Y. (2011). Diverse roles of TGF-β/Smads in renal fibrosis and inflammation. Int. J. Biol. Sci..

[26] Margadant C., Sonnenberg A. (2010). Integrin-TGF-β crosstalk in fibrosis, cancer and wound healing. Embo Rep..

[27] Hu H.H., Chen D.Q., Wang Y.N., Feng Y.L., Cao G., Vaziri N.D., Zhao Y.Y. (2018). New insights into TGF-β/Smad signaling in tissue fibrosis. Chem. Interact..

[28] Wick G., Grundtman C., Mayerl C., Wimpissinger T.F., Feichtinger J., Zelger B., Sgonc R., Wolfram D. (2013). The immunology of fibrosis. Annu. Rev. Immunol..

[29] Wick G., Backovic A., Rabensteiner E., Plank N., Schwentner C., Sgonc R. (2010). The immunology of fibrosis: Innate and adaptive responses. Trends Immunol..

[30] Schoberleitner I., Faserl K., Sarg B., Egle D., Brunner C., Wolfram D. (2023). Quantitative proteomic characterization of foreign body response towards silicone breast implants identifies chronological disease-relevant biomarker dynamics. Biomolecules.

[31] Siggelkow W., Gescher D.M., Siggelkow A., Klee D., Malik E., Rath W., Faridi A. (2004). In vitro analysis of modified surfaces of silicone breast implants. Int. J. Artif. Organs.

[32] Siggelkow W., Faridi A., Spiritus K., Klinge U., Rath W., Klosterhalfen B. (2003). Histological analysis of silicone breast implant capsules and correlation with capsular contracture. Biomaterials.

[33] Handel N., Jensen J.A., Black Q., Waisman J.R., Silverstein M.J. (1995). The fate of breast implants: A critical analysis of complications and outcomes. Plast. Reconstr. Surg..

[34] Kuehlmann B., Zucal I., Bonham C.A., Joubert L.M., Prantl L. (2021). SEM and TEM for identification of capsular fibrosis and cellular behavior around breast implants—A descriptive analysis. BMC Cell Biol..

[35] Prantl L., Schreml S., Fichtner-Feigl S., Pöppl N., Eisenmann-Klein M., Schwarze H., Füchtmeier B. (2007). Clinical and morphological conditions in capsular contracture formed around silicone breast implants. Plast. Reconstr. Surg..

[36] Colwell A.S., Taylor E.M. (2020). Recent advances in implant-based breast reconstruction. Plast. Reconstr. Surg..

[37] Frey J.D., Salibian A.A., Karp N.S., Choi M. (2019). Implant-based breast reconstruction: Hot topics, controversies, and new directions. Plast. Reconstr. Surg..

[38] Ricci J.A., Epstein S., Momoh A.O., Lin S.J., Singhal D., Lee B.T. (2017). A meta-analysis of implant-based breast reconstruction and timing of adjuvant radiation therapy. J. Surg. Res..

[39] Schoberleitner I., Augustin A., Egle D., Brunner C., Amort B., Zelger B., Brunner A., Wolfram D. (2023). Is it all about surface topography? An intra-individual clinical outcome analysis of two different implant surfaces in breast reconstruction. J. Clin. Med..

[40] Luvsannyam E., Patel D., Hassan Z., Nukala S., Somagutta M.R., Hamid P. (2020). Overview of risk factors and prevention of capsular contracture following implant-based breast reconstruction and cosmetic surgery: A systematic review. Cureus.

[41] Schoberleitner I., Faserl K., Tripp C.H., Pechriggl E.J., Sigl S., Brunner A., Zelger B., Hermann-Kleiter N., Baier L., Steinkellner T. (2024). Silicone implant surface microtopography modulates inflammation and tissue repair in capsular fibrosis. Front. Immunol..

[42] Lefkowitz D.L., Lefkowitz S.S. (2001). Macrophage-neutrophil interaction: A paradigm for chronic inflammation revisited. Immunol. Cell Biol..

[43] Sugimoto M.A., Vago J.P., Perretti M., Teixeira M.M. (2019). Mediators of the Resolution of the Inflammatory Response. Trends Immunol..

[44] Zhang M., Zhang S. (2020). T Cells in Fibrosis and Fibrotic Diseases. Front. Immunol..

[45] Safran T., Nepon H., Chu C.K., Winocour S., Murphy A.M., Davison P.G., Dionisopolos T., Vorstenbosch J. (2021). Healing, Inflammation, and Fibrosis: Current Concepts in Capsular Contracture: Pathophysiology, Prevention, and Management. Semin. Plast. Surg..

[46] Kim J.K., Scott E.A., Elbert D.L. (2005). Proteomic analysis of protein adsorption: Serum amyloid P adsorbs to materials and promotes leukocyte adhesion. J. Biomed. Mater. Res. A.

[47] Swartzlander M.D., Barnes C.A., Blakney A.K., Kaar J.L., Kyriakides T.R., Bryant S.J. (2015). Linking the foreign body response and protein adsorption to PEG-based hydrogels using proteomics. Biomaterials.

[48] Witherel C.E., Abebayehu D., Barker T.H., Spiller K.L. (2019). Macrophage and Fibroblast Interactions in Biomaterial-Mediated Fibrosis. Adv. Healthc. Mater..

[49] Greisler H.P. (1990). Interactions at the blood/material interface. Ann. Vasc. Surg..

[50] Faruq O., Pham N.C., Dönmez N., Nam S.Y., Heo C.Y. (2021). Functionalization of Silicone Surface with Drugs and Polymers for Regulation of Capsular Contracture. Polymers.

[51] Atiyeh B., Emsieh S. (2022). Effects of Silicone Breast Implants on Human Cell Types In Vitro: A Closer Look on Host and Implant. Aesth. Plast. Surg..

[52] Xing S., Santerre J.P., Labow R.S., Boynton E.L. (2002). The effect of polyethylene particle phagocytosis on the viability of mature human macrophages. J. Biomed. Mater. Res..

[53] Tavazzani F., Xing S., Waddell J.E., Smith D., Boynton E.L. (2005). In vitro interaction between silicone gel and human monocyte-macrophages. J. Biomed. Mater. Res. A.

[54] Wolfram D., Rabensteiner E., Grundtman C., Böck G., Mayerl C., Parson W., Almanzar G., Hasenöhrl C., Piza-Katzer H., Wick G. (2012). T regulatory cells and TH17 cells in peri-silicone implant capsular fibrosis. Plast. Reconstr. Surg..

[55] Cappellano G., Ploner C., Lobenwein S., Sopper S., Hoertnagl P., Mayerl C., Wick N., Pierer G., Wick G., Wolfram D. (2018). Immunophenotypic characterization of human T cells after in vitro exposure to different silicone breast implant surfaces. PLoS ONE.

[56] Backovic A., Wolfram D., Del-Frari B., Piza H., Huber L.A., Wick G. (2007). Simultaneous analysis of multiple serum proteins adhering to the surface of medical grade polydimethylsiloxane elastomers. J. Immunol. Methods.

[57] Wolfram D., Oberreiter B., Mayerl C., Soelder E., Ulmer H., Piza-Katzer H., Wick G., Backovic A. (2004). Cellular and molecular composition of fibrous capsules formed around silicone breast implants with special focus on local immune reactions. J. Autoimmun..

[58] Kuehlmann B., Burkhardt R., Kosaric N., Prantl L. (2018). Capsular fibrosis in aesthetic and reconstructive-cancer patients: A retrospective analysis of 319 cases. Clin. Hemorheol. Microcirc..

[59] Schoberleitner I., Baier L., Lackner M., Zenz L.-M., Coraça-Huber D.C., Ullmer W., Damerum A., Faserl K., Sigl S., Steinkellner T. (2024). Surface Topography, Microbial Adhesion, and Immune Responses in Silicone Mammary Implant-Associated Capsular Fibrosis. Int. J. Mol. Sci..

[60] Lin A.J., Karinja S.J., Bernstein J.L., Jin J., Toyoda Y., Miller A.J., Zanzonico P.B., Spector J.A. (2018). In Search of a Murine Model of Radiation-Induced Periprosthetic Capsular Fibrosis. Ann. Plast. Surg..

[61] Backovic A., Huang H.L., Del Frari B., Piza H., Huber L.A., Wick G. (2007). Identification and dynamics of proteins adhering to the surface of medical silicones in vivo and in vitro. J. Proteome Res..

[62] Wolfram D., Niederegger H., Piza H., Wick G. (2008). Altered systemic serologic parameters in patients with silicone mammary implants. Immunol. Lett..

[63] Doloff J.C., Veiseh O., de Mezerville R., Sforza M., Perry T.A., Haupt J., Jamiel M., Chambers C., Nash A., Aghlara-Fotovat S. (2021). The surface topography of silicone breast implants mediates the foreign body response in mice, rabbits, and humans. Nat. Biomed. Eng..

[64] ISAPS The Latest Global Survey from ISAPS Reports a Significant Rise in Aesthetic Surgery Worldwide. *Newswire* 2023. https://www.isaps.org.

[65] Alfano C., Mazzocchi M., Scuderi N. (2004). Mammary compliance: An objective measurement of capsular contracture. Aesthetic Plast. Surg..

[66] Henriksen T.F., Fryzek J.P., Hölmich L.R., McLaughlin J.K., Kjøller K., Høyer A.P., Olsen J.H., Friis S. (2005). Surgical intervention and capsular contracture after breast augmentation: A prospective study of risk factors. Ann. Plast. Surg..

[67] Henriksen T.F., Hölmich L.R., Fryzek J.P., Friis S., McLaughlin J.K., Høyer A.P., Kjøller K., Olsen J.H. (2003). Incidence and severity of short-term complications after breast augmentation: Results from a nationwide breast implant registry. Ann. Plast. Surg..

[68] Handel N., Cordray T., Gutierrez J., Jensen J.A. (2006). A long-term study of outcomes, complications, and patient satisfaction with breast implants. Plast. Reconstr. Surg..

[69] Eriksen C., Lindgren E.N., Frisell J., Stark B. (2012). A prospective randomized study comparing two different expander approaches in implant-based breast reconstruction: One stage versus two stages. Plast. Reconstr. Surg..

[70] Fischer J.P., Nelson J.A., Cleveland E., Sieber B., Rohrbach J.I., Serletti J.M., Kanchwala S. (2013). Breast reconstruction modality outcome study: A comparison of expander/implants and free flaps in select patients. Plast. Reconstr. Surg..

[71] Chisholm E.M., Marr S., Macfie J., Broughton A.C., Brennan T.G. (1986). Post-mastectomy breast reconstruction using the inflatable tissue expander. Br. J. Surg..

[72] McLaughlin J.K., Lipworth L., Murphy D.K., Walker P.S. (2007). The safety of silicone gel-filled breast implants: A review of the epidemiologic evidence. Ann. Plast. Surg..

[73] Chiu W.K., Fracol M., Feld L.N., Qiu C.S., Kim J.Y.S. (2021). Judging an expander by its cover: A propensity-matched analysis of the impact of tissue expander surface texture on first-stage breast reconstruction outcomes. Plast. Reconstr. Surg..

[74] Fairchild B., Ellsworth W., Selber J.C., Bogue D.P., Zavlin D., Nemir S., Checka C.M., Clemens M.W. (2020). Safety and efficacy of smooth surface tissue expander breast reconstruction. Aesthet. Surg. J..

[75] Lee K.T., Park H.Y., Jeon B.J., Mun G.H., Bang S.I., Pyon J.K. (2021). Does the textured-type tissue expander affect the outcomes of two-stage prosthetic breast reconstruction? A propensity score matching analysis between macrotextured and microtextured expanders. Plast. Reconstr. Surg..

[76] Mempin M., Hu H., Chowdhury D., Deva A., Vickery K. (2018). The A, B, and C’s of silicone breast implants: Anaplastic large cell lymphoma, biofilm, and capsular contracture. Materials.

[77] Zhang L., Haddouti E.M., Welle K., Burger C., Wirtz D.C., Schildberg F.A., Kabir K. (2020). The effects of biomaterial implant wear debris on osteoblasts. Front. Cell Dev. Biol..

[78] Groves A.M., Johnston C.J., Misra R.S., Williams J.P., Finkelstein J.N. (2016). Effects of IL-4 on pulmonary fibrosis and the accumulation and phenotype of macrophage subpopulations following thoracic irradiation. Int. J. Radiat. Biol..

[79] Trojanek J.B., Michałkiewicz J., Grzywa-Czuba R., Jańczyk W., Gackowska L., Kubiszewska I., Helmin-Basa A., Wierzbicka-Rucińska A., Szalecki M., Socha P. (2020). Expression of matrix metalloproteinases and their tissue inhibitors in peripheral blood leukocytes and plasma of children with nonalcoholic fatty liver disease. Mediat. Inflamm..

[80] Singh D., Rai V., Agrawal D.K. (2023). Regulation of collagen I and collagen III in tissue injury and regeneration. Cardiol. Cardiovasc. Med..

[81] Bachour Y., Verweij S.P., Gibbs S., Ket J.C.F., Ritt M.J.P.F., Niessen F.B., Mullender M.G. (2018). The aetiopathogenesis of capsular contracture: A systematic review of the literature. J. Plast. Reconstr. Aesthet. Surg..

[82] Spear S.L., Baker J.L. (1995). Classification of capsular contracture after prosthetic breast reconstruction. Plast. Reconstr. Surg..

[83] Sreejit G., Flynn M.C., Patil M., Krishnamurthy P., Murphy A.J., Nagareddy P.R. (2020). S100 family proteins in inflammation and beyond. Adv. Clin. Chem..

[84] Singh P., Ali S.A., Kalyuzhny E., Singh P., Ali S.A. (2022). Multifunctional role of S100 protein family in the immune system: An update. Cells.

[85] Gonzalez L.L., Garrie K., Turner M.D. (2020). Role of S100 proteins in health and disease. Biochim. Biophys. Acta (BBA) Mol. Cell Res..

[86] Sattar Z., Lora A., Jundi B., Railwah C., Geraghty P. (2021). The S100 Protein Family as Players and Therapeutic Targets in Pulmonary Diseases. Pulm. Med..

[87] Stevens W.G., Pacella S.J., Gear A.J.L., Freeman M.E., McWhorter C., Tenenbaum M.J., Stoker D.A. (2008). Clinical Experience With a Fourth-Generation Textured Silicone Gel Breast Implant: A Review of 1012 Mentor MemoryGel Breast Implants. Aesthet. Surg. J..

[88] Cole N.M. (2018). Consequences of the U.S. Food and Drug Administration-Directed Moratorium on Silicone Gel Breast Implants: 1992 to 2006. Plast. Reconstr. Surg..

[89] Coleman D.J., Foo I.T.H., Sharpe D.T. (1991). Textured or smooth implants for breast augmentation? A prospective controlled trial. Br. J. Plast. Surg..

[90] Wong C.H., Samuel M., Tan B.K., Song C. (2006). Capsular contracture in subglandular breast augmentation with textured versus smooth breast implants: A systematic review. Plast. Reconstr. Surg..

[91] Stevens W.G., Nahabedian M.Y., Calobrace M.B., Harrington J.L., Capizzi P.J., Cohen R., D’incelli R.C., Beckstrand M. (2013). Risk factor analysis for capsular contracture: A 5-year Sientra study analysis using round, smooth, and textured implants for breast augmentation. Plast. Reconstr. Surg..

[92] Lam M.C., Walgenbach-Brünagel G., Pryalukhin A., Vorhold J., Pech T., Kalff J.C., Kristiansen G., Walgenbach K.J. (2019). Management of Capsular Contracture in Cases of Silicone Gel Breast Implant Rupture with Use of Pulse Lavage and Open Capsulotomy. Aesthet. Plast. Surg..

[93] Atlan M., Kinney B.M., Perry T.A. (2020). Intra- and Inter-Shell Roughness Variability of Breast Implant Surfaces. Aesthet. Surg. J..

[94] Barr S., Hill E.W., Bayat A. (2017). Functional biocompatibility testing of silicone breast implants and a novel classification system based on surface roughness. J. Mech. Behav. Biomed. Mater..

[95] Zhang X.R., Chien P.N., Trinh X.T., Nam S.Y., Heo C.Y. (2022). Comparison of Formation of Capsule Among Different Breast Silicone Implants. In Vivo.

[96] Kyle D.J.T., Harvey A.G., Shih B., Tan K.T., Chaudhry I.H., Bayat A. (2013). Identification of molecular phenotypic descriptors of breast capsular contracture formation using informatics analysis of the whole genome transcriptome. Wound Repair Regen..

[97] Kuang J., Yan X., Genders A.J., Granata C., Bishop D.J. (2018). An overview of technical considerations when using quantitative real-time PCR analysis of gene expression in human exercise research. PLoS ONE.

[98] Ng M.T.H., Borst R., Gacaferi H., Davidson S., Ackerman J.E., Johnson P.A., Machado C.C., Reekie I., Attar M., Windell D. (2024). A single cell atlas of frozen shoulder capsule identifies features associated with inflammatory fibrosis resolution. Nat. Commun..

[99] Pang S., Yengo L., Nelson C.P., Bourier F., Zeng L., Li L., Kessler T., Erdmann J., Mägi R., Läll K. (2023). Genetic and modifiable risk factors combine multiplicatively in common disease. Clin. Res. Cardiol..

[100] McCarthy M.I., Abecasis G.R., Cardon L.R., Goldstein D.B., Little J., Ioannidis J.P.A., Hirschhorn J.N. (2008). Genome-wide association studies for complex traits: Consensus, uncertainty and challenges. Nat. Rev. Genet..

[101] McClellan J., King M.C. (2010). Genetic heterogeneity in human disease. Cell.

[102] Trastulla L., Dolgalev G., Moser S., Jiménez-Barrón L.T., Andlauer T.F.M., von Scheidt M., Budde M., Heilbronner U., Papiol S., Teumer A. (2024). Distinct genetic liability profiles define clinically relevant patient strata across common diseases. Nat. Commun..

[103] Barr S.P., Hill E.W., Bayat A. (2018). Novel Proteomic Assay of Breast Implants Reveals Proteins With Significant Binding Differences: Implications for Surface Coating and Biocompatibility. Aesthet. Surg. J..

[104] Staiano-Coico L., Higgins P.J., Schwartz S.B., Zimm A.J., Goncalves J. (2000). Wound fluids: A reflection of the state of healing. Ostomy Wound Manag..

[105] Harvey J., Mellody K.T., Cullum N., Watson R.E.B., Dumville J. (2022). Wound fluid sampling methods for proteomic studies: A scoping review. Wound Repair Regen..

[106] Hartman E., Wallblom K., van der Plas M.J.A., Petrlova J., Cai J., Saleh K., Kjellström S., Schmidtchen A. (2021). Bioinformatic Analysis of the Wound Peptidome Reveals Potential Biomarkers and Antimicrobial Peptides. Front. Immunol..

[107] Cialdai F., Risaliti C., Monici M. (2022). Role of fibroblasts in wound healing and tissue remodeling on Earth and in space. Front. Bioeng. Biotechnol..

[108] Monaco J.A.L., Lawrence W.T. (2003). Acute wound healing: An overview. Clin. Plast. Surg..

[109] Koh T.J., DiPietro L.A. (2011). Inflammation and wound healing: The role of the macrophage. Expert Rev. Mol. Med..

[110] Bautista-Hernández L.A., Gómez-Olivares J.L., Buentello-Volante B., Bautista-de Lucio V.M. (2017). Fibroblasts: The unknown sentinels eliciting immune responses against microorganisms. Eur. J. Microbiol. Immunol..

[111] Shinde A.V., Humeres C., Frangogiannis N.G. (2017). The role of α-smooth muscle actin in fibroblast-mediated matrix contraction and remodeling. Biochim. Biophys. Acta.

[112] Brazin J., Malliaris S., Groh B., Mehrara B., Hidalgo D., Otterburn D., Silver R.B., Spector J.A. (2014). Mast cells in the periprosthetic breast capsule. Aesthet. Plast. Surg..

[113] Noskovicova N., Hinz B., Pakshir P. (2021). Implant fibrosis and the underappreciated role of myofibroblasts in the foreign body reaction. Cells.

[114] Saalbach A., Klein C., Sleeman J., Sack U., Kauer F., Gebhardt C., Averbeck M., Anderegg U., Simon J.C. (2007). Dermal fibroblasts induce maturation of dendritic cells. J. Immunol..

[115] Langevin H.M., Nedergaard M., Howe A.K. (2013). Cellular control of connective tissue matrix tension. J. Cell Biochem..

[116] Correa-Gallegos D., Jiang D., Christ S., Ramesh P., Ye H., Wannemacher J., Gopal S.K., Yu Q., Aichler M., Walch A. (2019). Patch repair of deep wounds by mobilized fascia. Nature.

[117] Correa-Gallegos D., Jiang D., Rinkevich Y. (2021). Fibroblasts as confederates of the immune system. Immunol. Rev..

[118] Mescher A.L. (2017). Macrophages and fibroblasts during inflammation and tissue repair in models of organ regeneration. Regeneration.

[119] Veiseh O., Vegas A.J. (2019). Domesticating the foreign body response: Recent advances and applications. Adv. Drug Deliv. Rev..

[120] Akilbekova D., Bratlie K.M. (2015). Quantitative characterization of collagen in the fibrotic capsule surrounding implanted polymeric microparticles through second harmonic generation imaging. PLoS ONE.

[121] Pakshir P., Noskovicova N., Lodyga M., Son D.O., Schuster R., Goodwin A., Karvonen H., Hinz B. (2020). The myofibroblast at a glance. J. Cell Sci..

[122] Wynn T.A., Ramalingam T.R. (2012). Mechanisms of fibrosis: Therapeutic translation for fibrotic disease. Nat. Med..

[123] Lebleu V.S., Taduri G., O’Connell J., Teng Y., Cooke V.G., Woda C., Sugimoto H., Kalluri R. (2013). Origin and function of myofibroblasts in kidney fibrosis. Nat. Med..

[124] Higgins D.F. (2007). Hypoxia promotes fibrogenesis in vivo via HIF-1 stimulation of epithelial-to-mesenchymal transition. J. Clin. Investig..

[125] Darby I.A., Laverdet B., Bonté F., Desmoulière A. (2014). Fibroblasts and myofibroblasts in wound healing. Clin. Cosmet. Investig. Dermatol..

[126] Hinz B. (2016). The role of myofibroblasts in wound healing. Curr. Res. Transl. Med..

[127] Sun Y.B., Yang X., Qu G., Caruana G., Li J. (2016). The origin of renal fibroblasts/myofibroblasts and the signals that trigger fibrosis. Differentiation.

[128] Desjardins-Park H.E., Foster D.S., Longaker M.T. (2018). Fibroblasts and wound healing: An update. Regen. Med..

[129] Bainbridge P. (2013). Wound healing and the role of fibroblasts. J. Wound Care.

[130] Moyer K.E., Ehrlich H.P. (2013). Capsular contracture after breast reconstruction: Collagen fiber orientation and organization. Plast. Reconstr. Surg..

[131] Darby I.A., Hewitson T.D. (2007). Fibroblast differentiation in wound healing and fibrosis. Int. Rev. Cytol..

[132] Mirastschijski U., Schnabel R., Claes J., Schneider W., Ågren M.S., Haaksma C., Tomasek J.J. (2010). Matrix metalloproteinase inhibition delays wound healing and blocks the latent transforming growth factor-β1-promoted myofibroblast formation and function. Wound Repair Regen..

[133] Roeb E. (2018). Matrix metalloproteinases and liver fibrosis (translational aspects). Matrix Biol..

[134] Wong V.W., Gurtner G.C., Longaker M.T. (2013). Wound healing: A paradigm for regeneration. Mayo Clin. Proc..

[135] Tomasek J.J., Gabbiani G., Hinz B., Chaponnier C., Brown R.A. (2002). Myofibroblasts and mechano-regulation of connective tissue remodelling. Nat. Rev. Mol. Cell Biol..

[136] Sawant M., Hinz B., Schönborn K., Zeinert I., Eckes B., Krieg T., Schuster R. (2021). A story of fibers and stress: Matrix-embedded signals for fibroblast activation in the skin. Wound Repair Regen..

[137] Grinnell F., Petroll W.M. (2010). Cell motility and mechanics in three-dimensional collagen matrices. Annu. Rev. Cell Dev. Biol..

[138] Bernardo M.E., Fibbe W.E. (2013). Mesenchymal stromal cells: Sensors and switchers of inflammation. Cell Stem Cell.

[139] Hinz B., Lagares D. (2020). Evasion of apoptosis by myofibroblasts: A hallmark of fibrotic diseases. Nat. Rev. Rheumatol..

[140] Hinz B., McCulloch C.A., Coelho N.M. (2019). Mechanical regulation of myofibroblast phenoconversion and collagen contraction. Exp. Cell Res..

[141] Klingberg F., Hinz B., White E.S. (2013). The myofibroblast matrix: Implications for tissue repair and fibrosis. J. Pathol..

[142] Kang S., Kim J., Kim S., Wufuer M., Park S., Kim Y., Choi D., Jin X., Kim Y., Huang Y. (2020). Efficient reduction of fibrous capsule formation around silicone breast implants densely grafted with 2-methacryloyloxyethyl phosphorylcholine (MPC) polymers by heat-induced polymerization. Biomater. Sci..

[143] Wynn T.A. (2004). Fibrotic disease and the TH1/TH2 paradigm. Nat. Rev. Immunol..

[144] Plitas G., Rudensky A.Y. (2020). Regulatory T Cells in Cancer. Annu. Rev. Cancer Biol..

[145] Zhang X., Olsen N., Zheng S.G. (2020). The progress and prospect of regulatory T cells in autoimmune diseases. J. Autoimmun..

[146] Sakaguchi S., Mikami N., Wing J.B., Tanaka A., Ichiyama K., Ohkura N. (2020). Regulatory T Cells and Human Disease. Annu. Rev. Immunol..

[147] Motwani K., Peters L.D., Vliegen W.H., El-Sayed A.G., Seay H.R., Lopez M.C., Baker H.V., Posgai A.L., Brusko M.A., Perry D.J. (2020). Human Regulatory T Cells From Umbilical Cord Blood Display Increased Repertoire Diversity and Lineage Stability Relative to Adult Peripheral Blood. Front. Immunol..

[148] Roncarolo M.G., Gregori S., Bacchetta R., Battaglia M., Gagliani N. (2018). The Biology of T Regulatory Type 1 Cells and Their Therapeutic Application in Immune-Mediated Diseases. Immunity.

[149] Savage P.A., Klawon D.E.J., Miller C.H. (2020). Regulatory T Cell Development. Annu. Rev. Immunol..

[150] Shevach E.M. (2011). Biological functions of regulatory T cells. Adv. Immunol..

[151] Taylor A.E., Carey A.N., Kudira R., Lages C.S., Shi T., Lam S., Karns R., Simmons J., Shanmukhappa K., Almanan M. (2018). Interleukin 2 promotes hepatic regulatory T cell responses and protects from biliary fibrosis in murine sclerosing cholangitis. Hepatology.

[152] Lee G.R. (2018). The balance of Th17 versus Treg cells in autoimmunity. Int. J. Mol. Sci..

[153] Boks N.P., Norde W., van der Mei H.C., Busscher H.J. (2008). Forces involved in bacterial adhesion to hydrophilic and hydrophobic surfaces. Microbiology.

[154] Yong Y., Qiao M., Chiu A., Fuchs S., Liu Q., Pardo Y., Worobo R., Liu Z., Ma M. (2019). Conformal hydrogel coatings on catheters to reduce biofouling. Langmuir.

[155] Smithmyer M.E., Sawicki L.A., Kloxin A.M. (2014). Hydrogel scaffolds as in vitro models to study fibroblast activation in wound healing and disease. Biomater. Sci..

[156] Erathodiyil N., Chan H.M., Wu H., Ying J.Y. (2020). Zwitterionic polymers and hydrogels for antifouling applications in implantable devices. Mater. Today.

[157] Zhang L., Cao Z., Bai T., Carr L., Ella-Menye J.R., Irvin C., Ratner B.D., Jiang S. (2013). Zwitterionic hydrogels implanted in mice resist the foreign-body reaction. Nat. Biotechnol..

[158] Foroushani F.T., Dzobo K., Khumalo N.P., Zamora Mora V., de Mezerville R., Bayat A. (2022). Advances in surface modifications of the silicone breast implant and impact on its biocompatibility and biointegration. Biomater. Res..

[159] Macdonald W., Campbell P., Fisher J., Wennerberg A. (2004). Variation in surface texture measurements. J. Biomed. Mater. Res. Part B Appl. Biomater..

[160] Lampin M., Warocquier-Clérout R., Legris C., Degrange M., Sigot-Luizard M.F. (1997). Correlation between substratum roughness and wettability, cell adhesion, and cell migration. J. Biomed. Mater. Res..

[161] Atlan M., Bigerelle M., Larreta-garde V., Hindié M., Hedén P. (2016). Characterization of breast implant surfaces, shapes, and biomechanics: A comparison of high cohesive anatomically shaped textured silicone breast implants from three different manufacturers. Aesthetic Plast. Surg..

[162] Prasad B.R., Brook M.A., Smith T., Zhao S., Chen Y., Sheardown H., D’souza R., Rochev Y. (2010). Controlling cellular activity by manipulating silicone surface roughness. Colloids Surf. B Biointerfaces.

[163] Barth K.A., Waterfield J.D., Brunette D.M. (2013). The effect of surface roughness on RAW 264.7 macrophage phenotype. J. Biomed. Mater. Res. Part A.

[164] Brigaud I., Garabédian C., Bricout N., Pieuchot L., Ponche A., Deltombe R., Delille R., Atlan M., Bigerelle M., Anselme K. (2020). Surface texturization of breast implants impacts extracellular matrix and inflammatory gene expression in asymptomatic capsules. Plast. Reconstr. Surg..

[165] Doloff J.C., Veiseh O., Vegas A.J., Tam H.H., Farah S., Ma M., Li J., Bader A., Chiu A., Sadraei A. (2017). Colony stimulating factor-1 receptor is a central component of the foreign body response to biomaterial implants in rodents and non-human primates. Nat. Mater..

[166] Anderson J.M., Rodriguez A., Chang D.T. (2008). Foreign body reaction to biomaterials. Semin. Immunol..

[167] Sussman E.M., Halpin M.C., Muster J., Moon R.T., Ratner B.D. (2014). Porous implants modulate healing and induce shifts in local macrophage polarization in the foreign body reaction. Ann. Biomed. Eng..

[168] Wolf M.T., Dearth C.L., Ranallo C.A., LoPresti S.T., Carey L.E., Daly K.A., Brown B.N., Badylak S.F. (2014). Macrophage polarization in response to ECM coated polypropylene mesh. Biomaterials.

[169] Klopfleisch R., Jung F. (2017). The pathology of the foreign body reaction against biomaterials. J. Biomed. Mater. Res. Part A.

[170] Dondossola E., Holzapfel B.M., Alexander S., Filippini S., Hutmacher D.W., Friedl P. (2016). Examination of the foreign body response to biomaterials by nonlinear intravital microscopy. Nat. Biomed. Eng..

[171] Miron R.J., Bosshardt D.D. (2018). Multinucleated giant cells: Good guys or bad guys?. Tissue Eng. Part B Rev..

[172] Spiller K.L., Anfang R.R., Spiller K.J., Ng J., Nakazawa K.R., Daulton J.W., Vunjak-Novakovic G. (2014). The role of macrophage phenotype in vascularization of tissue engineering scaffolds. Biomaterials.

[173] Mooney J.E., Rolfe B.E., Osborne G.W., Sester D.P., Van Rooijen N., Campbell G.R., Hume D.A., Campbell J.H. (2010). Cellular plasticity of inflammatory myeloid cells in the peritoneal foreign body response. Am. J. Pathol..

[174] Smith T.D., Nagalla R.R., Chen E.Y., Liu W.F. (2017). Harnessing macrophage plasticity for tissue regeneration. Adv. Drug Deliv. Rev..

[175] Martin K.E., García A.J. (2021). Macrophage phenotypes in tissue repair and the foreign body response: Implications for biomaterial-based regenerative medicine strategies. Acta Biomater..

[176] Graney P.L., Lurier E.B., Spiller K.L. (2018). Biomaterials and bioactive factor delivery systems for the control of macrophage activation in regenerative medicine. ACS Biomater. Sci. Eng..

[177] Bonner J.C. (2004). Regulation of PDGF and its receptors in fibrotic diseases. Cytokine Growth Factor Rev..

[178] McNally A.K., Anderson J.M. (2015). Phenotypic expression in human monocyte-derived interleukin-4-induced foreign body giant cells and macrophages in vitro: Dependence on material surface properties. J. Biomed. Mater. Res. A.

[179] Abaricia J.O., Farzad N., Heath T.J., Simmons J., Morandini L., Olivares-Navarrete R. (2021). Control of innate immune response by biomaterial surface topography, energy, and stiffness. Acta Biomater..

[180] Love R.J., Jones K.S. (2013). The recognition of biomaterials: Pattern recognition of medical polymers and their adsorbed biomolecules. J. Biomed. Mater. Res. A.

[181] Altieri D.C., Mannucci P.M., Capitanio A.M. (1986). Binding of fibrinogen to human monocytes. J. Clin. Investig..

[182] Aiyelabegan H.T., Sadroddiny E. (2017). Fundamentals of protein and cell interactions in biomaterials. Biomed. Pharmacother..

[183] Sheikh Z., Brooks P.J., Barzilay O., Fine N., Glogauer M. (2015). Macrophages, foreign body giant cells and their response to implantable biomaterials. Materials.

[184] Helming L., Gordon S. (2009). Molecular mediators of macrophage fusion. Trends Cell Biol..

[185] Zhang Y., Cheng X., Jansen J.A., Yang F., van den Beucken J.J.J.P. (2019). Titanium surfaces characteristics modulate macrophage polarization. Mater. Sci. Eng. C.

[186] Shayan M., Padmanabhan J., Morris A.H., Cheung B., Smith R., Schroers J., Kyriakides T.R. (2018). Nanopatterned bulk metallic glass-based biomaterials modulate macrophage polarization. Acta Biomater..

[187] Mohiuddin M., Pan H.A., Hung Y.C., Huang G.S. (2012). Control of growth and inflammatory response of macrophages and foam cells with nanotopography. Nanoscale Res. Lett..

[188] Padmanabhan J., Augelli M.J., Cheung B., Kinser E.R., Cleary B., Kumar P., Wang R., Sawyer A.J., Li R., Schwarz U.D. (2016). Regulation of cell-cell fusion by nanotopography. Sci. Rep..

[189] Previtera M.L., Sengupta A. (2015). Substrate stiffness regulates proinflammatory mediator production through TLR4 activity in macrophages. PLoS ONE.

[190] Irwin E.F., Saha K., Rosenbluth M., Gamble L.J., Castner D.G., Healy K.E. (2008). Modulus-dependent macrophage adhesion and behavior. J. Biomater. Sci. Polym. Ed..

[191] Bizjak M., Selmi C., Praprotnik S., Bruck O., Perricone C., Ehrenfeld M., Shoenfeld Y. (2015). Silicone implants and lymphoma: The role of inflammation. J. Autoimmun..

[192] Yoon H.M., Lee C.Y., Song W.J. (2023). Axillary silicone lymphadenopathy caused by gel bleeding with intact silicone breast implants: A case report. Arch. Aesthetic Plast. Surg..

[193] Fleury E.D.F.C., Fleury E.D.F.C. (2020). Silicone induced granuloma of breast implant capsule (SIGBIC) diagnosis: Breast magnetic resonance (BMR) sensitivity to detect silicone bleeding. PLoS ONE.

[194] Dziubek M., Laurent R., Bonapace-Potvin M., Gaboury L., Danino M.A. (2023). Silicone particles in capsules around breast implants: Establishment of a new pathological methodology to assess the number of particles around breast implants. Ann. Chir. Plast. Esthet..

[195] Danino M.A., Dziubek M., Dalfen J., Bonapace-Potvin M., Gaboury L., Giot J.P., Laurent R. (2024). Silicone particles in capsules around breast implants: An investigation into currently available implants in North America. Aesthetic Surg. J..

[196] Hallab N.J., Samelko L., Hammond D. (2019). The inflammatory effects of breast implant particulate shedding: Comparison with orthopedic implants. Aesthetic Surg. J..

[197] Fleury E., Nimir C., Salum G.D. (2021). The breast tumor microenvironment: Could silicone breast implant elicit breast carcinoma?. Breast Cancer Target Ther..

[198] Hallab N.J., Samelko L., Hammond D. (2021). Particulate debris released from breast implant surfaces is highly dependent on implant type. Aesthetic Surg. J..

[199] Bekerecioglu M., Onat A.M., Tercan M., Buyukhatipoglu H., Karakok M., Isik D., Bulut O. (2008). The association between silicone implants and both antibodies and autoimmune diseases. Clin. Rheumatol..

[200] Suh L.J., Khan I., Kelley-Patteson C., Mohan G., Hassanein A.H., Sinha M. (2022). Breast implant-associated immunological disorders. J. Immunol. Res..

[201] van Haasterecht L., Zada L., Schmidt R.W., de Bakker E., Barbé E., Leslie H.A., Vethaak A.D., Gibbs S., de Boer J.F., Niessen F.B. (2020). Label-free stimulated Raman scattering imaging reveals silicone breast implant material in tissue. J. Biophotonics.

[202] Gristina A.G., Naylor P., Myrvik Q. (1998). Infections from biomaterials and implants: A race for the surface. Med. Prog. Technol..

[203] Belay E.D., Schonberger L.B., Brown P., Priola S.A., Chesebro B., Will R.G., Asher D.M. (2010). Disinfection and sterilization of prion-contaminated medical instruments. Infect. Control Hosp. Epidemiol..

[204] Rutala W.A., Weber D.J. (2001). New disinfection and sterilization methods. Emerg. Infect. Dis..

[205] Rutala W.A., Weber D.J. (2010). Guideline for disinfection and sterilization of prion-contaminated medical instruments. Infect. Control Hosp. Epidemiol..

[206] Donlan R.M. (2002). Biofilms: Microbial life on surfaces. Emerg. Infect. Dis..

[207] Vestby L.K., Grønseth T., Simm R., Nesse L.L. (2020). Bacterial biofilm and its role in the pathogenesis of disease. Antibiotics.

[208] Malte H. (1999). The DLVO theory in microbial adhesion. Colloids Surf. B Biointerfaces.

[209] Berne C., Ellison C.K., Ducret A., Brun Y.V. (2018). Bacterial adhesion at the single-cell level. Nat. Rev. Microbiol..

[210] Costerton J.W. (1999). Bacterial biofilms: A common cause of persistent infections. Science.

[211] Fuqua W.C., Winans S.C., Greenberg E.P. (1994). Quorum sensing in bacteria: The LuxR-LuxI family of cell density-responsive transcriptional regulators. J. Bacteriol..

[212] Rumbaugh K.P., Sauer K. (2020). Biofilm dispersion. Nat. Rev. Microbiol..

[213] Josse J., Laurent F., Diot A. (2017). Staphylococcal adhesion and host cell invasion: Fibronectin-binding and other mechanisms. Front. Microbiol..

[214] Herman-Bausier P., Valotteau C., Pietrocola G., Rindi S., Alsteens D., Foster T.J., Speziale P., Dufrêne Y.F. (2016). Mechanical strength and inhibition of the Staphylococcus aureus collagen-binding protein cna. mBio.

[215] Walker J.N., Pinkner C.L., Pinkner J.S., Hultgren S.J., Myckatyn T.M. (2019). The detection of bacteria and matrix proteins on clinically benign and pathologic implants. Plast. Reconstr. Surg. Glob. Open.

[216] Tamboto H., Vickery K., Deva A.K. (2010). Subclinical (biofilm) infection causes capsular contracture in a porcine model following augmentation mammaplasty. Plast. Reconstr. Surg..

[217] Barbieri R., Pesce M., Franchelli S., Baldelli I., De Maria A., Marchese A. (2015). Phenotypic and genotypic characterization of Staphylococci causing breast peri-implant infections in oncologic patients. BMC Microbiol..

[218] Miller K.E., Hontanilla B., Cabello A., Marre D., Armendariz L., Leiva J. (2016). The effect of late infection and antibiotic treatment on capsular contracture in silicone breast implants: A rat model. J. Plast. Reconstr. Aesthet. Surg..

[219] Anderson J.M. (2019). Biocompatibility and bioresponse to biomaterials. Principles of Regenerative Medicine.

[220] Anderson J.M., McNally A.K. (2011). Biocompatibility of implants: Lymphocyte/macrophage interactions. Semin. Immunopathol..

